# Prophage-encoded methyltransferase drives adaptation of community-acquired methicillin-resistant *Staphylococcus aureus*

**DOI:** 10.1172/JCI177872

**Published:** 2025-07-22

**Authors:** Robert J. Ulrich, Magdalena Podkowik, Rebecca Tierce, Irnov Irnov, Gregory Putzel, Nora M. Samhadaneh, Keenan A. Lacey, Daiane Boff, Sabrina M. Morales, Sohei Makita, Theodora K. Karagounis, Erin E. Zwack, Chunyi Zhou, Randie H. Kim, Karl Drlica, Alejandro Pironti, Harm van Bakel, Victor J. Torres, Bo Shopsin

**Affiliations:** 1 Department of Medicine and; 2 Antimicrobial-Resistant Pathogens Program, New York University (NYU) Grossman School of Medicine, New York, New York, USA.; 3 Division of Comparative Medicine, NYU Langone Health, New York, New York, USA.; 4 Department of Microbiology,; 5 Department of Pathology, and; 6 Ronald O. Perelman Department of Dermatology, NYU Grossman School of Medicine, New York, New York, USA.; 7 Department of Microbiology, Biochemistry & Molecular Genetics, New Jersey Medical School, Rutgers University, Newark, New Jersey, USA.; 8 Department of Biochemistry and Molecular Pharmacology, NYU Grossman School of Medicine, New York, New York, USA.; 9 Department of Genetics and Genomic Sciences,; 10 Department of Microbiology,; 11 Icahn Genomics Institute, Icahn School of Medicine at Mount Sinai, New York, New York, USA.; 12 Department of Host-Microbe Interactions, St. Jude Children’s Research Hospital, Memphis, Tennessee, USA.

**Keywords:** Infectious disease, Microbiology, Bacterial infections, Epigenetics, Molecular biology

## Abstract

We recently described the evolution of a community-acquired methicillin-resistant *Staphylococcus aureus* (CA-MRSA) USA300 variant responsible for an outbreak of skin and soft tissue infections. Acquisition of a mosaic version of the Φ11 prophage (mΦ11) that increases skin abscess size was an early step in CA-MRSA adaptation that primed the successful spread of the clone. The present study shows how prophage mΦ11 exerts its effect on virulence for skin infection without encoding known toxin or fitness genes. Abscess size and skin inflammation were associated with DNA methylase activity of an mΦ11-encoded adenine methyltransferase (designated *pamA*). *pamA* increased expression of fibronectin-binding protein A (*fnbA*; FnBPA), and inactivation of *fnbA* eliminated the effect of *pamA* on abscess virulence without affecting strains lacking *pamA*. Thus, *fnbA* is a *pamA*-specific virulence factor. Mechanistically, *pamA* was shown to promote biofilm formation in vivo in skin abscesses, a phenotype linked to FnBPA’s role in biofilm formation. Collectively, these data reveal a critical mechanism — epigenetic regulation of staphylococcal gene expression — by which phage can regulate virulence to drive adaptive leaps by *S*. *aureus*.

## Abstract 2

A prophage-encoded adenine methyltransferase from an outbreak variant of methicillin-resistant *Staphylococcus aureus* increased skin infection virulence by upregulating fibronectin binding protein A

## Graphical abstract

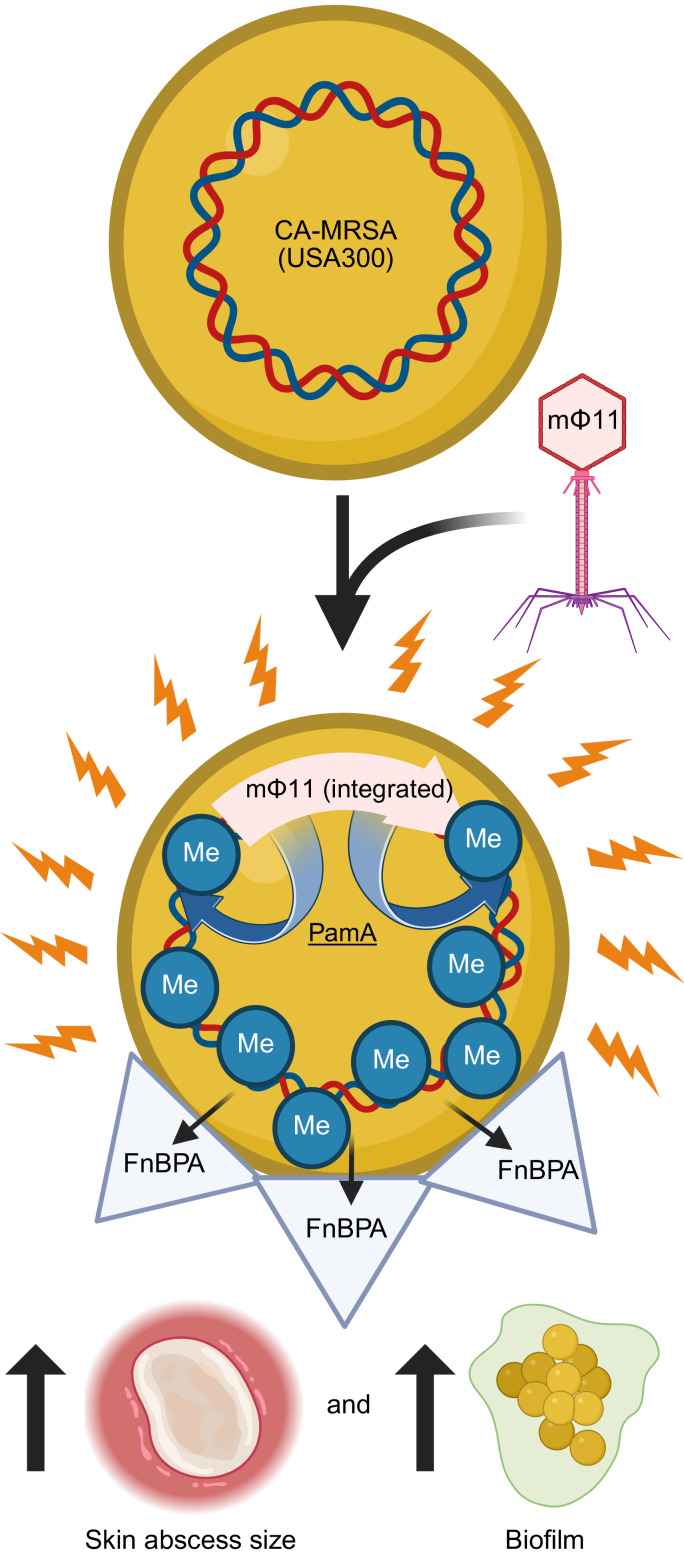

## Introduction

Community-acquired methicillin-resistant *Staphylococcus aureus* (CA-MRSA) lineage USA300 is a major cause of skin and soft tissue infection in the United States, and clonal variants that cause outbreaks have become public health emergencies (1–3). The spectrum of adaptive changes that arise during the course of CA-MRSA dissemination is likely to identify genetic pathways critical for bacterial pathogenesis in vivo (4). We recently described a genotypic cluster of CA-MRSA-USA300 that emerged in Brooklyn, New York (USA300-BKV), which was uniquely positioned to offer insight into properties of emerging CA-MRSA strains (5). The persistence of the Brooklyn disease cluster enabled us to use phylogenetic analysis and experimental assays to identify a unique prophage that promoted large skin abscesses. That pathogen advantage primed USA300-BKV for successful spread, thereby facilitating the subsequent emergence of resistance to topical antimicrobials (5). Until now, it was unknown how the Brooklyn cluster–associated prophage, which is a mosaic variant of the well-known *S*. *aureus* generalized transducing phage Φ11 (referred to as mΦ11), enhanced virulence during skin infection.

### S.

*aureus* strains often carry multiple prophages (6–8), which are primary drivers of *S*. *aureus* evolution, diversity, and virulence (9–12). To date, studies of prophage-mediated virulence in *S*. *aureus* have primarily focused on prophage-encoded toxin and fitness genes. One example is the prophage-encoded Panton-Valentine leucocidin, which is associated with skin abscesses across all *S*. *aureus* lineages, including USA300 (13–16). In contrast, the Brooklyn cluster–associated mΦ11 is similar to most prophages in that it lacks a known virulence factor, and its insertion does not disrupt a chromosomal virulence gene (5). Thus, new pathogenic mechanisms are expected to derive from genetic and phenotypic analysis that define mΦ11 components driving enhanced skin abscess virulence.

In the present work, we report that the methylase activity of an mΦ11-encoded DNA methyltransferase, which has been named *pamA* for phage adenine methyltransferase A, is necessary and sufficient for the enhanced skin abscess phenotype observed with the USA300-BKV clone. Moreover, we found that *pamA* rewires the bacterial transcriptional program, resulting in a marked increase in the expression and production of fibronectin-binding protein A (*fnbA*; FnBPA), as determined by RNA-Seq and global proteomics. *fnbA* was necessary for the *pamA*-associated skin abscess phenotype, which was in turn associated with increased production of biofilms. Collectively, the data demonstrate how phage can modify DNA to enhance USA300 virulence by altering the expression of core genome-encoded virulence factors, thereby increasing the fitness of epidemic clones and driving leaps in adaptation.

## Results

### Genetic deletions define the mΦ11 gene(s) responsible for increased skin infection virulence.

**Figure 1:**
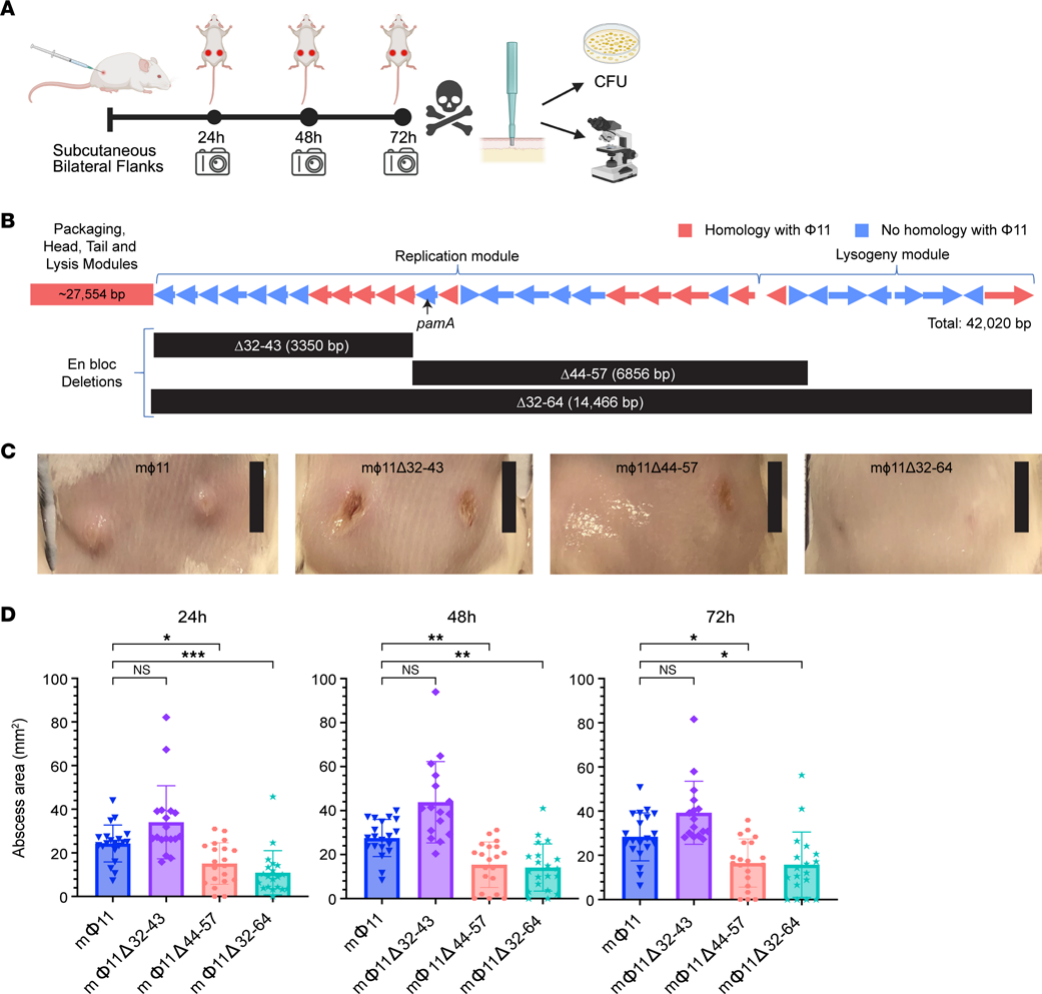
Effect of en bloc deletions on the mΦ11-mediated skin abscess phenotype. (**A**) Skin infection workflow. Created with BioRender.com. (**B**) Map of mΦ11 in strain USA300-BKV, adapted with permission from Copin et al. (5), with en bloc deletion locations. Arrows indicate predicted ORFs and the direction of the transcription of genes within the unique mΦ11 modules. Homologous (red) and nonhomologous (blue) ORFs are shown, compared with prototypical Φ11. Black arrow indicates *pamA*. Black bars beneath the gene map correspond to the gene blocks deleted from the indicated strain. (**C**) Representative images of skin abscesses 72 hours after subcutaneous infection with the indicated strains. Scale bar (black): 1 cm. (**D**) Skin abscess infections with en bloc deletion mutants. Skin abscess area at the indicated times after infection with approximately 10^7^ bacterial CFU of LAC* lysogens containing mΦ11 (blue, *n* = 20, strain BS989), mΦ11Δ32–43 (purple, *n* = 16–18, strain RU47), mΦ11Δ44–57 (salmon, *n* = 20, strain RU108), or mΦ11Δ32–64 (cyan, *n* = 18–20, strain RU42). Data are pooled from 2 independent experiments and represent mean ± SD. Statistical significance was determined by Kruskal-Wallis and Dunn’s tests, *P ≤ 0.05, **P ≤ 0.01, ***P ≤ 0.001.

Prophage mΦ11 promotes USA300-mediated tissue damage by increasing skin abscess size during murine infection (Supplemental Figure 1A; supplemental material available online with this article; https://doi.org/10.1172/JCI177872DS1), as previously reported (5). Often, hypervirulent strains of *S*. *aureus* will exhibit differences in growth rates (17), secreted protein production (18), and/or transcriptional profiles (19–21). However, strain USA300 LAC* harboring mΦ11 did not show significantly altered in vitro growth kinetics, hemolysis patterns, biofilm production, exoprotein profiles, or transcript levels of non-mΦ11 genes compared with the parental USA300 LAC*, as determined by RNA-Seq analysis (Supplemental Figure 1, B–F). Therefore, results of the in vitro analyses did not correlate with the increased virulence demonstrated by mΦ11-containing strains during skin infection. These data suggest that an in vivo signal(s) is required for mΦ11-associated virulence (22). Thus, in vivo models of infection are required to identify mechanisms underlying mΦ11-mediated virulence (Figure 1A).

Annotation of the mosaic portion of mΦ11 failed to identify known virulence factors (5). Consequently, we constructed 3 en bloc deletions within the mosaic region of mΦ11 to identify candidate gene(s) (Figure 1B). The deletions were confirmed using whole genome sequencing (Supplemental Figure 2). As expected, deletion of the entire mosaic region containing genes in the replication and lysogeny modules (Δ32–64) eliminated the mΦ11 skin abscess phenotype in mice (Figure 1, C and D). Although deletion of an upstream fragment (Δ32–43) had no impact on abscess size, deletion of the center gene block (Δ44–57) eliminated the skin abscess phenotype (Figure 1, C and D). These data localized skin abscess candidates to 14 genes (genes 44–57) in mΦ11 for further analysis. Of the 14 genes, 8 gene sequences were unrelated to prototypical Φ11 or other known prophages and therefore were considered promising candidates for further analysis (Supplemental Table 1).

### An mΦ11-encoded adenine methyltransferase is responsible for increased skin infection virulence.

Examination of the 8 potential virulence genes identified a methyltransferase that was absent in wild-type Φ11 (Supplemental Table 1). The mΦ11-encoded adenine methyltransferase (*pamA*) shares amino acid sequence homology with DNA adenine methyltransferases (*dam*) (5), so-called orphan methyltransferases that are not paired with a cognate restriction endonuclease and therefore do not form an obvious restriction-modification system. DNA adenine methyltransferases act independently to regulate gene expression and bacterial replication (23–25). They have also been implicated in prophage-mediated pathogenicity of an outbreak strain of *E*. *coli* (26). Thus, *pamA* represented a promising candidate gene as a virulence regulator during skin infection.

**Figure 2:**
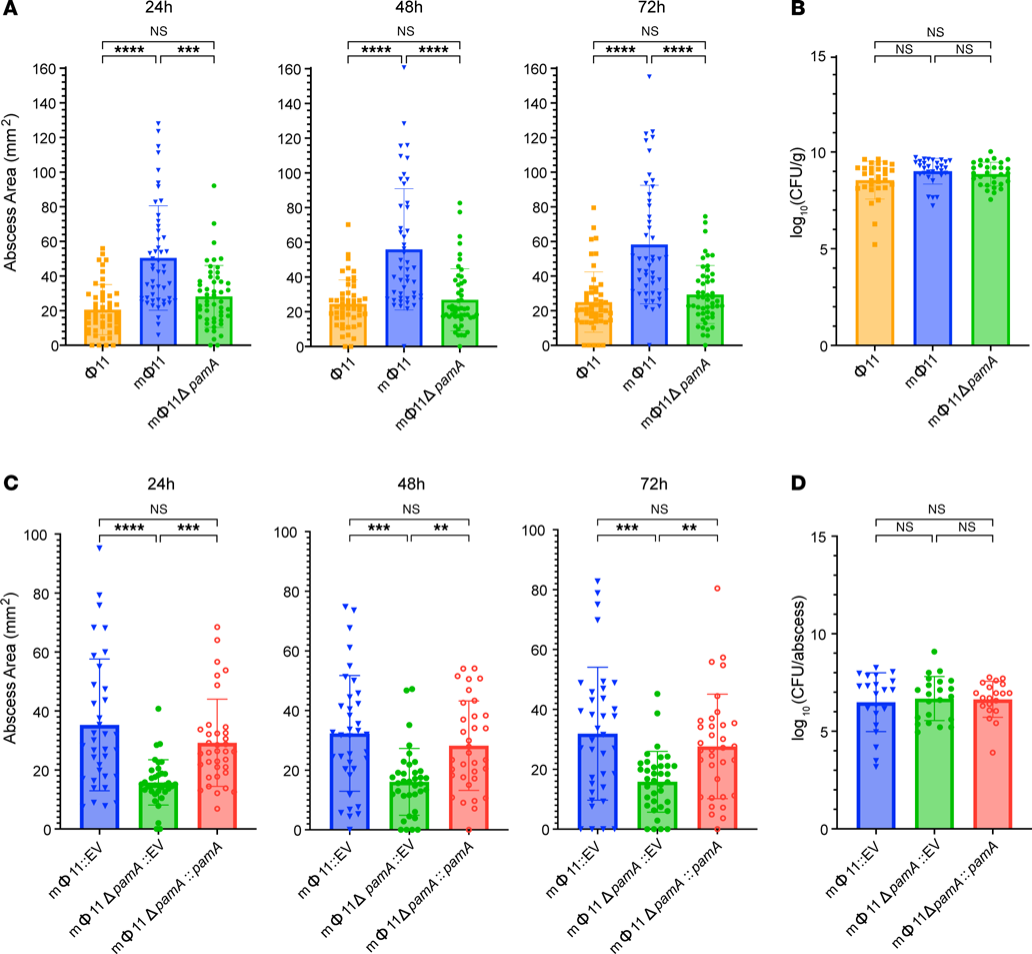
mΦ11 phage adenine methyltransferase increases skin abscess size without affecting tissue bacterial burden. (**A**) Effect of *pamA* on the mΦ11 skin abscess phenotype. Abscess area at the indicated times after infection with approximately 1.5 × 10^7^ bacterial CFU of LAC* containing Φ11 (orange, *n* = 50 abscesses, strain BS990), mΦ11 (blue, *n* = 48–50 abscesses, strain BS989), or mΦ11Δ*pamA* (green, *n* = 50 abscesses, strain RU39). Data are pooled from 4 independent experiments and represent mean ± SD. Statistical significance determined by Kruskal-Wallis and Dunn’s tests, ***P ≤ 0.001, ****P ≤ 0.0001. (**B**) *pamA* skin abscess bacterial burden. Skin abscesses from **A** with LAC* containing Φ11 (orange, *n* = 30 abscesses, strain BS990), mΦ11 (blue, *n* = 30 abscesses, strain BS989), or mΦ11Δ*pamA* (green, *n* = 30 abscesses, strain RU39) were harvested at 72 hours and abscess CFU enumerated. Data represent mean ± SD. Statistical significance determined by Kruskal-Wallis and Dunn’s tests. (**C**) Effect of *pamA* complementation on abscess size. Abscess area at the indicated times after infection with approximately 1 × 10^7^ bacterial CFU of LAC* containing mΦ11:EV (blue, *n* = 36 abscesses, strain RU138), mΦ11Δ*pamA*:EV (green, *n* = 36 abscesses, strain RU128), or mΦ11Δ*pamA*:*pamA* (red, *n* = 34–36 abscesses, strain RU131). EV, empty vector. Data are pooled from 4 independent experiments and represent mean ± SD. Statistical significance determined by Kruskal-Wallis and Dunn’s tests, **P ≤ 0.01, ***P ≤ 0.001, ****P ≤ 0.0001. (**D**) Bacterial burden in abscesses. Skin abscesses from **C** of LAC* containing mΦ11:EV (blue, *n* = 22 abscesses, strain RU138), mΦ11Δ*pamA*:EV (green, *n* = 22 abscesses, strain RU128), and mΦ11Δ*pamA*:*pamA* (red, *n* = 20 abscesses, strain RU131) were harvested at 72 hours and CFU enumerated. CFU/abscess is shown due to missing abscess weights during one of the replicate experiments. With the available weight-adjusted data, we found no significant differences between strains (not shown). Data represent mean ± SD. Statistical significance determined by Kruskal-Wallis and Dunn’s tests.

To determine whether *pamA* is necessary for the enhanced skin abscess phenotype, we engineered an in-frame, unmarked *pamA* deletion in a USA300 LAC* mΦ11 lysogen (mΦ11Δ*pamA*). Sanger and whole genome sequencing confirmed the deletion and absence, respectively, of adventitious secondary mutations in mΦ11Δ*pamA* (Supplemental Figure 3). Infection of mice with mΦ11Δ*pamA* resulted in a nearly identical average skin abscess size compared with that of the control Φ11 lysogen (Figure 2A), suggesting that *pamA* was necessary for increased virulence. Complementation, by integration of constitutively expressed *pamA* into the staphylococcal chromosome in a single copy using the *S*. *aureus* pathogenicity island (SapI) *att* site (27), verified that *pamA* is responsible for the skin abscess phenotype (Figure 2C). We did not observe a difference in bacterial burden at 72 hours after infection (Figure 2, B and D) or dermonecrosis area (Supplemental Figure 5A), as previously reported for comparisons between mΦ11 and Φ11 lysogens (5). Collectively, these data demonstrated that mΦ11-encoded *pamA* was required for increased skin abscess size in mice.

### pamA increases CA-MRSA skin abscess size irrespective of other mΦ11 genes and in non-USA300 backgrounds.

**Figure 3:**
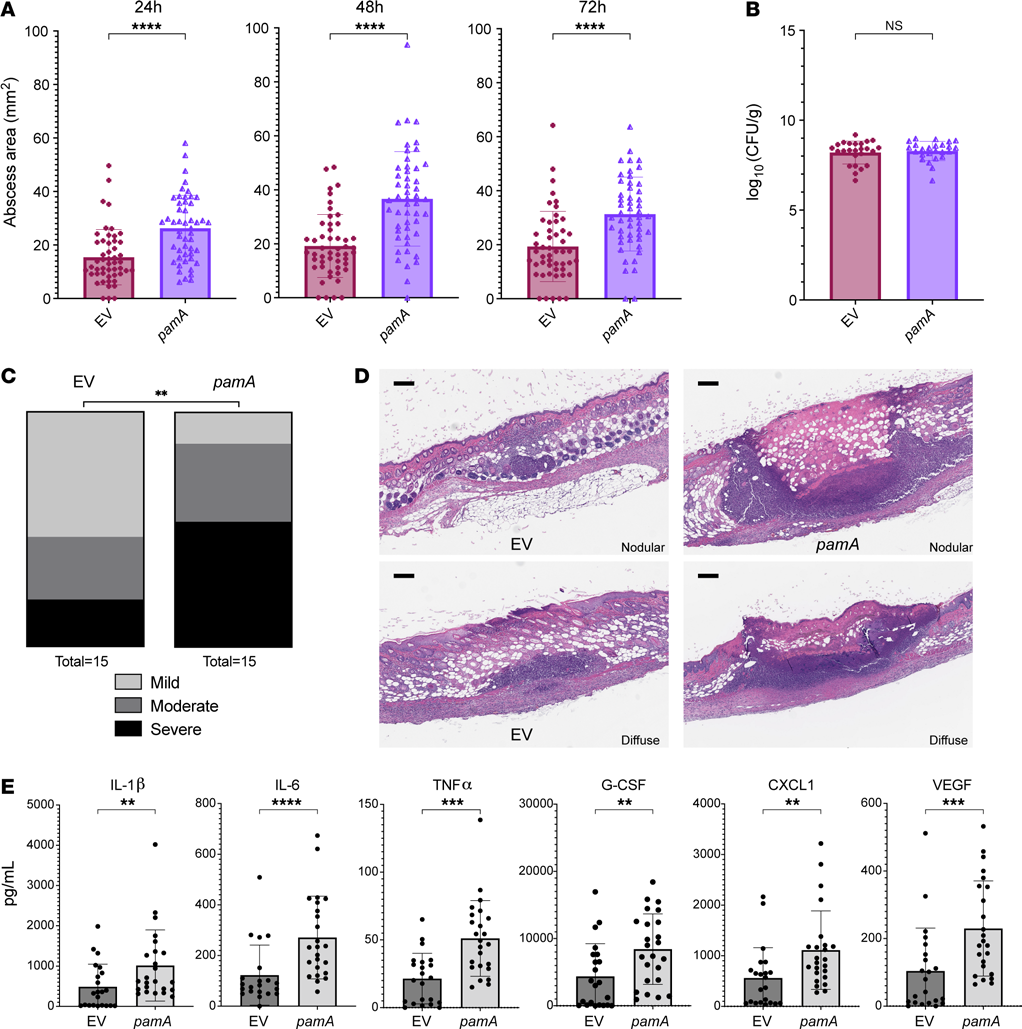
*pamA* increases skin abscess size and inflammation in the absence of mΦ11. (**A**) Effect of *pamA* on skin abscess size. Abscess area at the indicated times after infection with approximately 1 × 10^7^ bacterial CFU of LAC* with empty vector (EV) (maroon, *n* = 50 abscesses, strain RU129) or constitutively expressed *pamA* (purple, *n* = 50 abscesses, strain RU121) integrated into the chromosome in single copy. Data are pooled from 4 independent experiments and represent mean ± SD. Statistical significance was determined by Mann-Whitney test, ****P ≤ 0.0001. (**B**) Effect of *pamA* on CFU recovered from skin abscesses. Skin abscesses (*n* = 25 abscesses per strain) from 2 independent infections in **A** were harvested at 72 hours and CFU enumerated. Data represent mean ± SD. Statistical significance determined by Mann-Whitney test. (**C**) Effect of *pamA* on skin inflammation. Biopsies of skin abscess (*n* = 15 per strain, pooled from 2 independent experiments) 72 hours after infection with LAC* containing *pamA* (strain RU121) or EV control (strain RU129) were H&E stained and inflammatory burden was graded by a blinded dermatopathologist. Statistical significance determined by χ^2^ test (*P* = 0.0014). (**D**) Representative images of skin abscess biopsies from **C**. One image from each strain is presented according to dermatopathologist architecture classification as nodular (above) or diffuse (below). (**E**) Effect of *pamA* on local proinflammatory and vascular proliferation cytokines. Skin abscess biopsies from 3 independent experiments of LAC* with *pamA* (*n* = 24 abscesses, strain RU121) or EV control (*n* = 22 abscesses, strain RU129) were homogenized, and levels of the indicated cytokines were measured. Data represent mean ± SD. Statistical significance was determined by Mann-Whitney test, **P ≤ 0.01, ***P ≤ 0.001, ****P ≤ 0.0001.

Next, we hypothesized that *pamA* expression would increase CA-MRSA virulence independent of other mΦ11 genes. Indeed, wild-type USA300 LAC*-expressing *pamA* (LAC*:*pamA*) produced larger abscesses than an empty vector control strain (LAC*:EV), as shown in Figure 3A. A maximal increase in abscess area of 91% was observed at 48 hours after infection. Even during an extended 10 days of postinfection monitoring, despite an overall decrease in abscess size, LAC*:*pamA* produced significantly larger skin abscesses than those of LAC*:EV (Supplemental Figure 4A). Therefore, *pamA* was sufficient to increase CA-MRSA skin virulence. As with mΦ11 lysogens, LAC*:*pamA* did not affect bacterial CFU recovered from skin abscesses (Figure 3B and Supplemental Figure 4B) or lesion dermonecrosis area (Supplemental Figure 5B), indicating that pamA increases abscess size through a mechanism independent of bacterial burden or toxin production, respectively. Considering these findings, we posited that *pamA* increases abscess size by increasing tissue inflammation. To test this, we compared skin abscess histology and murine cytokine production of LAC*:*pamA* and LAC*:EV at 72 hours after infection. Skin abscess inflammatory burden (Figure 3, C and D) and proinflammatory cytokine/chemokine production (Figure 3E) were increased in LAC*:*pamA* skin abscesses compared with control LAC*:EV. Together, these data demonstrated that insertion of *pamA* into a USA300 LAC* background without the surrounding mΦ11 genes was sufficient to increase local tissue inflammation and thereby skin abscess size.

Although *pamA* was discovered in USA300, a strain within clonal complex (CC) 8, we wanted to test whether the *pamA* virulence effects are relevant in other clinical MRSA strain backgrounds. While mΦ11 failed to lysogenize non-CC8 strains, suggesting that mΦ11 is lineage specific to USA300/CC8, we continued our investigation by inserting the *pamA* expression vector into the chromosome of a CC1 MRSA strain from our clinical collection (BVED028). This strain was selected because it represents the same lineage as the well-characterized CA-MRSA prototype MW2, but unlike MW2, its resistance profile was compatible with our vector system. Even in the CC1 background, *pamA* increased abscess size to a similar degree as our findings in a CC8 (LAC*) background without affecting CFU recovery (Supplemental Figure 6). These data demonstrate that the virulence effects of *pamA* could extend to MRSA strains from different genetic backgrounds.

### pamA-associated skin abscess virulence depends on methyltransferase activity.

**Figure 4:**
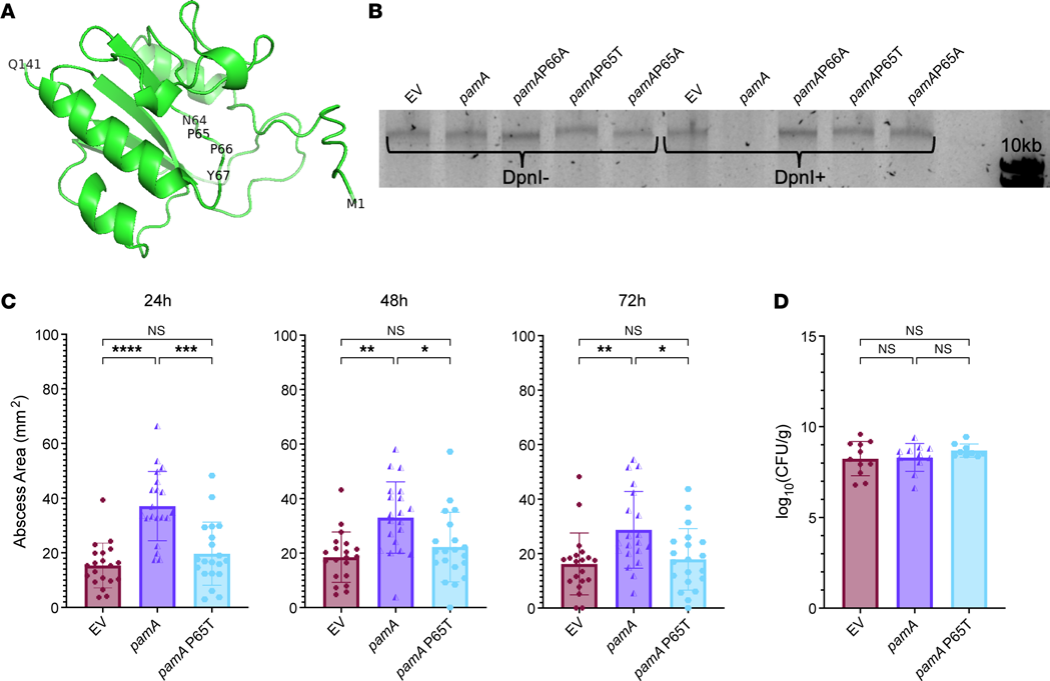
The *pamA*-mediated skin abscess phenotype depends on the methylase activity of PamA. (**A**) Predicted structure of mΦ11 PamA. Amino acid backbone represented in green, with N-terminus (M1), C-terminus (Q141), and putative active site (N64, P65, P66, Y67) highlighted. Generated by AlphaFold, visualized using PyMol Molecular Graphics System, version 2.5.2 (Schrödinger, LLC). (**B**) Effect of PamA point mutants on methylase activity. Genomic DNA was isolated from LAC* strains containing the indicated *pamA* alleles and digested with DpnI (DpnI+) or PBS control (DpnI–), then visualized on a 1% agarose gel. The analysis confirms that PamA methylates at the predicted GATC site and that PamA point mutants lack methylation activity. EV, empty vector. (**C**) Skin abscess size. Abscess area of LAC* with EV (maroon, *n* = 20 abscesses, strain RU129), *pamA* (purple, *n* = 20 abscesses, strain RU121), and *pamA*P65T (cyan, *n* = 20 abscesses, strain RU162) at the indicated time points after skin infection with approximately 1 × 10^7^ CFU of bacteria per abscess. Data are pooled from 2 independent experiments and represent mean ± SD. Statistical significance was determined by Kruskal-Wallis and Dunn’s tests, *P ≤ 0.05, **P ≤ 0.01, ***P ≤ 0.001, ****P ≤ 0.0001. (**D**) Bacterial burden in abscesses. Skin abscesses from infections in panel **C** (*n* = 9–11 abscesses per strain) were harvested at 72 hours and CFU enumerated. Data represent mean ± SD. Statistical significance was determined by Kruskal-Wallis and Dunn’s tests.

To determine whether *pamA* increases skin abscess virulence through methylase activity, we identified the conserved Dam active site (NPPY) in PamA (Figure 4A) and individually introduced several point mutations in residues previously reported to inactivate methyltransferase activity (28). To confirm that the PamA point mutants were inactive, we digested genomic DNA with the restriction endonuclease DpnI, an enzyme that digests at the methylated target of Dam (GATC) (29). As expected, *pamA*-containing strains, but not those with point mutations in *pamA*, were susceptible to DpnI digestion (Figure 4B).

For in vivo studies, we used the *S*. *aureus* strain containing *pamA*P65T, since this substitution exhibited the most substantial decrease in Dam methylation activity (28). Consistent with the hypothesis that the methylation activity of PamA contributes to the increased abscess size, LAC*:*pamA*P65T produced abscesses that were 32%–47% smaller than LAC*:*pamA* abscesses and similar in size to the LAC*:EV control (Figure 4C). The P65T amino acid change eliminated the *pamA*-mediated large-size skin abscess size phenotype without affecting tissue bacterial burden in the underlying tissues (Figure 4D). We conclude that the DNA methylation activity of PamA increases abscess virulence.

### Identification of genes involved in pamA-mediated virulence.

**Figure 5:**
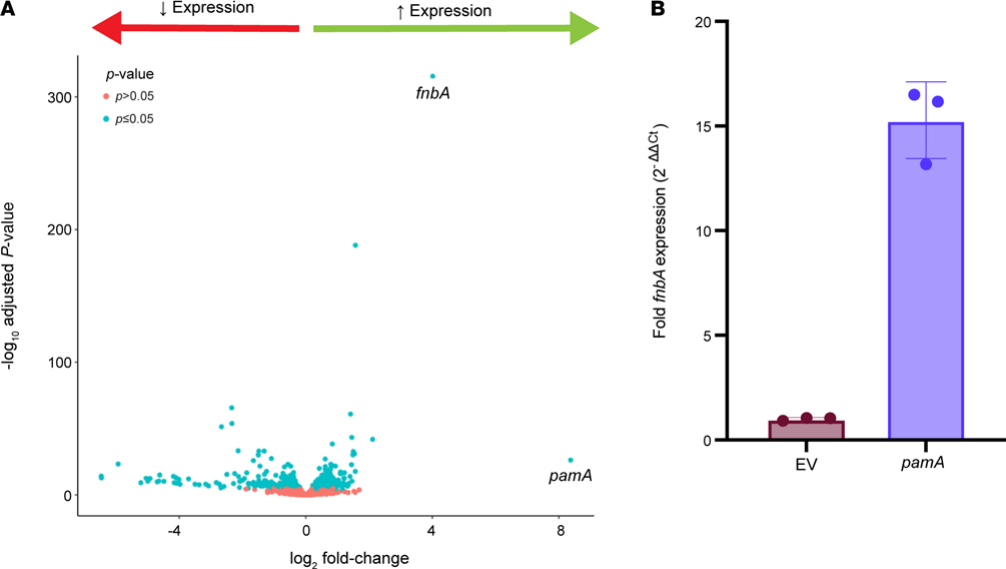
*pamA* induces widespread transcriptional changes, including a large increase in the expression of fibronectin-binding protein A (*fnbA*; FnBPA). (**A**) Whole genome transcriptome. Volcano plot of RNA-Seq data comparing LAC* strains containing *pamA* (*n* = 3 biological replicates, strain RU121) or empty vector (EV) control (*n* = 2 biological replicates, strain RU129) after 5 hours of growth in RPMI media. Data points to the right of 0 (green arrow) represent upregulated genes in LAC*:*pamA* and data points to the left of 0 (red arrow) represent downregulated genes in LAC*:*pamA*; *pamA* and *fnbA* are highlighted. Blue data points represent genes that achieved statistical significance (*P* ≤ 0.05); pink data points indicate genes that did not. (**B**) Effect of *pamA* on *fnbA* expression. Quantitative real-time PCR of *fnbA* expression in LAC* strains containing *pamA* or EV control. Strains were grown and prepared in the same manner as **A**. Data represent mean SD of 3 biological replicates.

We proceeded to investigate whether *pamA* epigenetically regulates bacterial gene(s) that result in the hyper-abscess phenotype. Notably, *pamA* is constitutively expressed in LAC*:*pamA*, allowing us to bypass the in vivo induction needed to produce mΦ11-related phenotypes. The need for constitutive expression to explore in vitro *pamA* methylation effects was exemplified by DpnI digest of LAC*:*pamA* and parental mΦ11 strains, in which the mΦ11 strains showed minimal *pamA* methylation changes (Supplemental Figure 7). This likely contributes to the absence of in vitro phenotypes in the parental mΦ11 strain (Supplemental Figure 1, B–F) and informed us that LAC*:*pamA* is required to investigate the effects of *pamA* methylation on global transcription. Thus, we performed RNA-Seq with LAC*:*pamA* and LAC*:EV strains during exponential growth in nutrient-restrictive (RPMI) medium chosen to resemble nutrient availability under infectious conditions in human plasma (30). LAC*:*pamA* induced widespread transcriptional changes in CA-MRSA compared with the LAC*:EV control, with 483 genes differentially expressed (232 overexpressed, 250 underexpressed, adjusted *P* < 0.05) (Figure 5A). The most significantly upregulated gene in LAC*:*pamA* compared with LAC*:EV encodes fibronectin-binding protein A (*fnbA*; FnBPA) (Figure 5A). Quantitative reverse transcriptase PCR (qRT-PCR) confirmed a 15-fold increase in *fnbA* transcription in the LAC*:*pamA* strain compared with LAC*:EV (Figure 5B). FnBPA is an *S*. *aureus* cell wall–anchored protein that binds adhesive matrix molecules, increasing *S*. *aureus* invasion into nonprofessional phagocytic cells (31–33). FnBPA also induces platelet aggregation (34), promotes biofilm formation (35–37), and has been implicated as a virulence factor in endocarditis (38), sepsis (39), implant infections (40), and skin and soft tissue infections (41). Collectively, these observations suggest that *fnbA* plays a role in *pamA*-mediated virulence.

### pamA enhances biofilm production in vitro and in vivo by increasing FnBPA.

**Figure 6:**
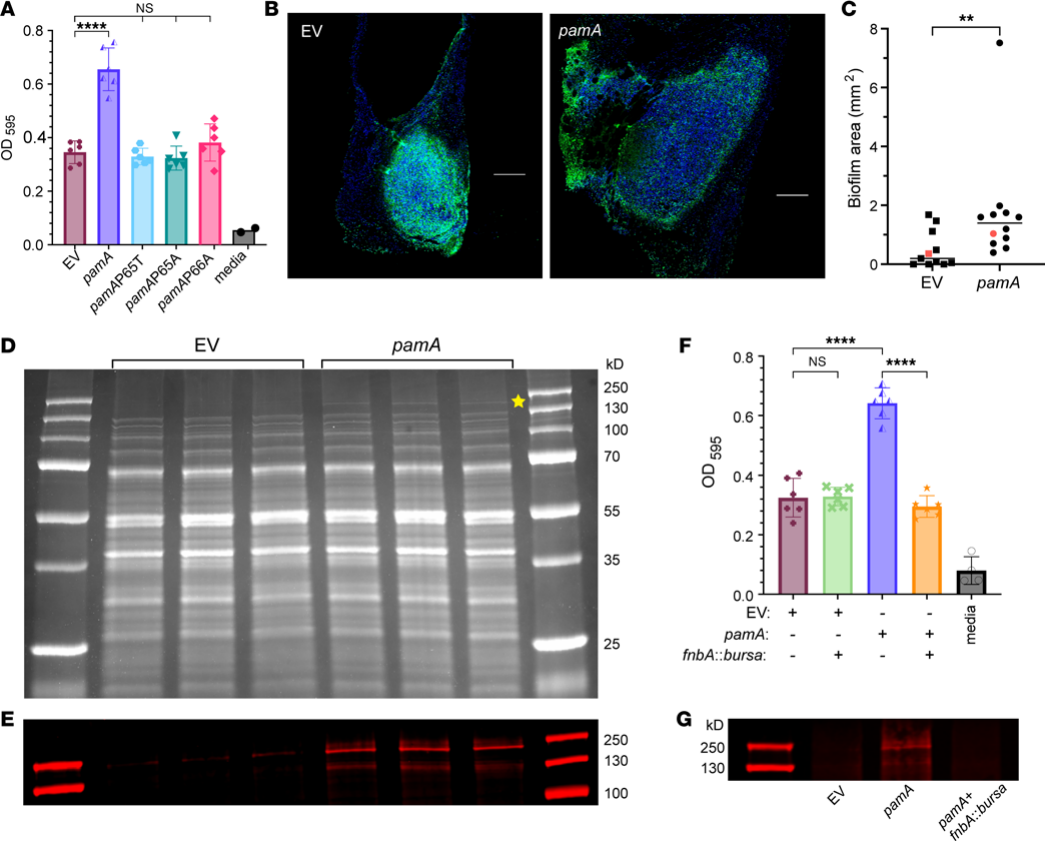
*pamA* methylase increases biofilm production through *fnbA* (FnBPA). (**A**) Effect of *pamA* methylase on biofilm production. In vitro biofilm quantified by OD after static growth for 24 hours by LAC* strains with the indicated *pamA* alleles integrated into the chromosome. EV, empty vector. Data represent mean ± SD and are pooled from 2 independent experiments. Statistical significance was determined by ANOVA with Tukey’s test, ****P ≤ 0.0001. (**B**) Effect of *pamA* on abscess biofilm. Representative images of skin abscess tissue stained with DAPI (blue) and 5-methylcytosine (5-mC, green) 72 hours after infection with approximately 1 × 10^7^ CFU of LAC* containing *pamA* (strain RU121) or EV (strain RU129). Scale bar: 200 μm. (**C**) Biofilm area of LAC* containing *pamA* (*n* = 12 abscesses, strain RU121) or EV (*n* = 11 abscesses, strain RU129) quantified as the difference between DAPI (total extracellular DNA) and 5-mC (eukaryotic host extracellular DNA) (48). Red data points correspond to representative images in **B**. Data are pooled from 2 independent experiments; individual results are shown in Supplemental Figure 9A. Statistical significance was determined by Mann-Whitney test, **P ≤ 0.01. The difference remained significant (*P* = 0.007) after removal of the *pamA* strain outlier (Supplemental Figure 9B). (**D**) Cell wall proteins. Cell wall–associated proteins from biofilms of LAC* strains containing *pamA* or EV (3 biological replicates each) were separated by SDS-PAGE. Gel image represents 2 independent experiments. Yellow star = band of interest. (**E**) Identification of FnBPA bands. Western blot of cell wall–associated protein bands from **D** using polyclonal anti-FnBPA. (**F**) Biofilm production. In vitro biofilms from LAC* strains containing the indicated genetic changes quantified by OD. Data represent mean ± SD of 6 biological replicates per strain, pooled from 2 independent experiments. (**G**) FnBPA production. Western blot of cell wall–associated proteins during in vitro biofilm production by the indicated strains.

The upregulation of *fnbA* expression observed in *pamA-*containing strains, coupled with its association with biofilm-related infections (42), suggest that *pamA* increases the formation of biofilms. Indeed, we found that LAC*:*pamA* nearly doubled biofilm production compared with LAC*:EV in an in vitro biofilm assay (Figure 6A). This phenotype reverted to LAC*:EV when *pamA* contained an inactivating point mutation (Figure 6A). Scanning electron microscopy of in vitro biofilms revealed a more robust bacterial aggregation and architecture, while SYTOX staining demonstrated increased extracellular DNA in the LAC*:*pamA* biofilms compared with LAC*:EV (Supplemental Figure 8). Thus, the methylase activity of *pamA* increased biofilm production.

### S.

*aureus* biofilm has traditionally been associated with device-related infections (43, 44), endocarditis (45), and osteomyelitis (46). However, we and others have found that biofilms also form during *S*. *aureus* deep tissue abscess infections (47, 48). Additionally, skin abscess size correlates with in vitro biofilm formation with *S*. *aureus* (49). To determine whether *pamA*-mediated biofilms form in vivo, we quantified biofilm production in skin abscess tissue of LAC*:*pamA* and LAC*:EV control strains by immunofluorescent staining of extracellular bacterial DNA (48), an abundant component of *S*. *aureus* biofilms (50). LAC*:*pamA* strains produced 6-fold more biofilm compared with the LAC*:EV control (Figure 6, B and C, and Supplemental Figure 9). Thus, *pamA* stimulated biofilm production in skin abscesses, supporting the idea that *pamA*-mediated biofilm production is important for pathogenesis of the skin abscess phenotype.

To test whether FnBPA production was increased in *pamA*-associated biofilms, we compared levels of FnBPA in biofilms from LAC*:*pamA* and LAC*:EV control strains. Bacterial cell wall–associated proteins from in vitro biofilms, as determined by SDS-PAGE, are shown in Figure 6D. A distinct, high–molecular weight protein band was observed to be more abundant in LAC*:*pamA* compared with control strain LAC*:EV; there was otherwise considerable similarity in the distributions of the corresponding bands obtained from the 2 strains. Consistent with our transcriptional data, the high–molecular weight protein band was identified as FnBPA by mass spectrometry (Supplemental Figure 10) and confirmed by Western blot (Figure 6E).

To determine whether FnBPA was responsible for increased biofilm production, we compared biofilm formation in an *fnbA-*inactivated mutant of LAC*:*pamA* (LAC*:*pamA* plus *fnbA*:*bursa*) and a control strain carrying an empty vector (LAC*:EV plus *fnbA*:*bursa*). The results showed that LAC*:*pamA* plus *fnbA*:*bursa* phenocopied the biofilm production of the LAC*:EV strain (Figure 6F); therefore, the *fnbA* inactivation reversed the biofilm-enhancing effect of *pamA*. Western blot of cell wall–associated proteins from biofilm-associated bacteria confirmed that LAC*:*pamA* increased FnBPA production (Figure 6G). Thus, *pamA* increases biofilm production in USA300 LAC* by increasing production of FnBPA.

### pamA increases skin infection virulence through fnbA.

**Figure 7:**
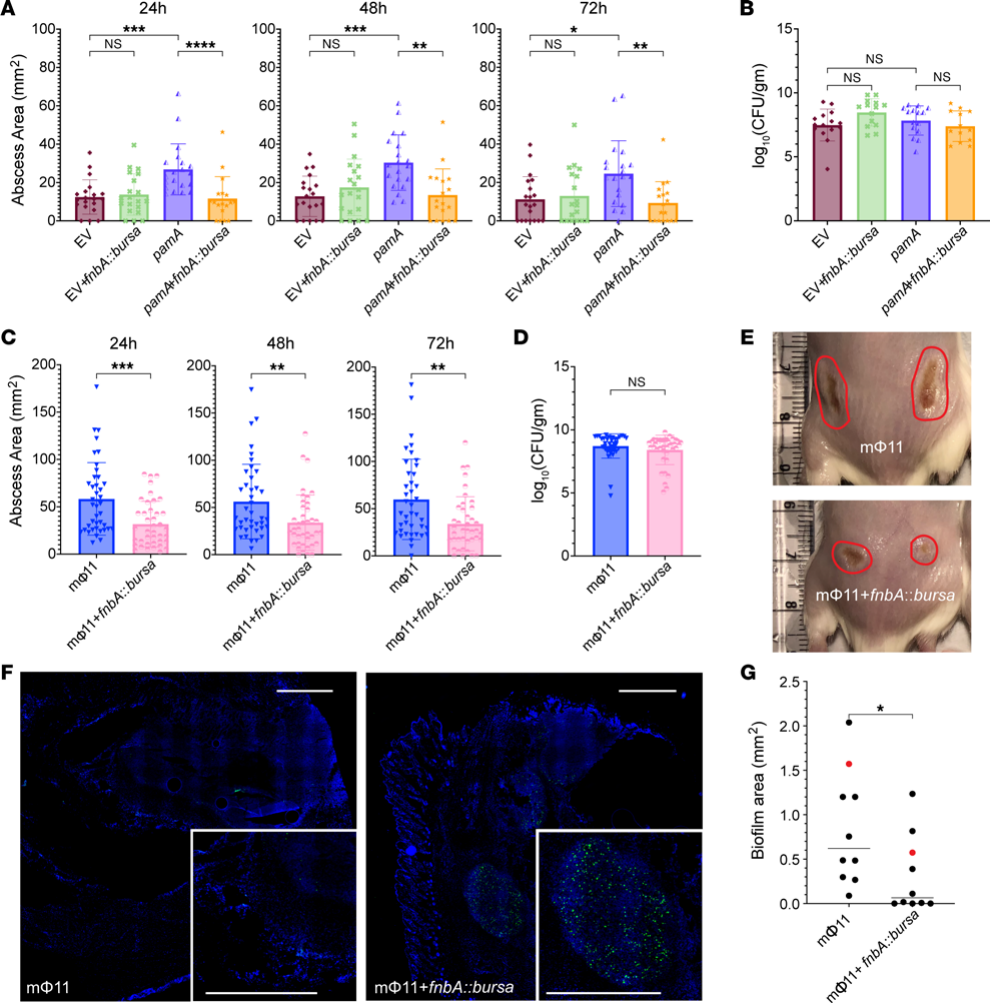
*fnbA* deficiency decreases *pamA*-mediated skin abscess size and biofilm production in vivo. (**A**) Effect of *fnbA* on the *pamA*-mediated abscesses. Abscess area at the indicated times after skin infection with approximately 1 × 10^7^ bacterial CFU of LAC* with EV (maroon, *n* = 20 abscesses, strain RU129), EV+*fnbA:bursa* (green, *n* = 18–20 abscesses, strain RU170), *pamA* (purple, *n* = 20 abscesses, strain RU121), or *pamA*+*fnbA:bursa* (orange, *n* = 18 abscesses, strain RU169). Data are pooled from 2 independent experiments and represent mean ± SD. (**B**) Bacterial burden in abscesses. Abscesses from **A** (*n* = 14–15 per strain) were harvested and CFU enumerated 72 hours after infection. Data represent mean ± SD. (**C**) Effect of *fnbA* on the mΦ11-mediated abscesses. Abscess area at the indicated times after infection with approximately 1 × 10^7^ bacterial CFU of LAC* containing mΦ11 (blue, *n* = 40 abscesses, strain BS989) or mΦ11+*fnbA:bursa* (pink, *n* = 40 abscesses, strain RU171). Data are pooled from 4 independent experiments and represent mean ± SD. (**D**) Skin abscesses from **C** were harvested 72 hours after infection and CFU enumerated. Data represent mean ± SD. (**E**) Representative images 72 hours after infection with the indicated strains; abscess area circled. (**F**) Effect of *fnbA* on biofilm formation in mΦ11-mediated skin abscesses. Representative images of abscess tissue stained for DAPI (blue) and 5-methylcytosine (5-mC, green) 72 hours after infection with approximately 1 × 10^7^ CFU of LAC* containing mΦ11 (strain BS989, left image) or mΦ11 with *fnbA:bursa* (strain RU171, right image). The corner inset is magnified abscess area. Scale bars: 1,000 μm. (**G**) Biofilm area of the strains from **F** (*n* = 10 abscesses each) quantified as the difference between DAPI and 5-mC (48). Red data points correspond to representative images in **F**. Statistical significance for **A** and **B** was determined by Kruskal-Wallis and Dunn’s tests. Statistical significance in the remaining panels was determined by Mann-Whitney test, *P ≤ 0.05, **P ≤ 0.01, ***P ≤ 0.001, ****P ≤ 0.0001.

To investigate whether *fnbA* was responsible for *pamA*-associated skin abscess virulence, we compared LAC*:*pamA* plus *fnbA*:*bursa* mutant and control LAC*:*pamA* strains. Strain LAC*:*pamA* plus *fnbA:bursa* produced 56%–62% smaller abscesses than strain LAC*:*pamA* (Figure 7A). At the same time, inactivation of *fnbA* did not affect abscess size in the LAC*:EV control, indicating that the upregulation of *fnbA* by *pamA* is required for the observed phenotype. We found no difference in abscess tissue bacterial CFU related to the presence of *pamA* or *fnbA* (Figure 7B), supporting the idea that *fnbA* is necessary for the increased inflammatory response seen in LAC*:*pamA*.

To ensure that the in vivo phenotype was specific to *pamA*, and not an artifact of *pamA* overexpression in LAC*:*pamA*, we compared mice infected with an *fnbA-*inactivated mutant of mΦ11 (LAC*/mΦ11:*fnbA*:*bursa*) with mice infected with control strain LAC*/mΦ11. The results showed that *fnbA* was critical for the increased skin abscess size observed with mΦ11-containing strains (Figure 7, C–E). Furthermore, disruption of *fnbA* significantly decreased skin abscess biofilm production in the mΦ11 strain (Figure 7, F and G). In the mΦ11 strain, *pamA* expression was under its native control, so these results indicate that overexpression alone cannot explain the increased skin infection virulence or abscess biofilm production demonstrated in LAC*:*pamA*. We conclude that LAC*:*pamA* can provide insights into the role of *pamA* in the virulence of mΦ11-containing strains and that *fnbA* is an essential downstream virulence factor in the *pamA* regulatory pathway underlying increased virulence during skin infection.

## Discussion

The present findings, in conjunction with previous molecular epidemiology observations (5), indicate that the mechanism by which mΦ11 primed the epidemic USA300 Brooklyn clone for success is via a phage adenine methyltransferase (*pamA*) that increases bacterial virulence during skin abscess infection. We discovered that *pamA* mediates this increase by rewiring the strain through epigenetic modifications that induce expression of *fnbA*, a virulence factor essential for the enhanced virulence of mΦ11-containing USA300. Interestingly, we found that *fnbA* was not a skin abscess virulence factor in the absence of *pamA*. FnBPA was, in turn, associated with increased abscess inflammation and an increased ability to form biofilms. Successful spread of the Brooklyn clone after acquisition of mΦ11 is tied to subsequent selection of antimicrobial resistance (5). Thus, analysis of the USA300-phage interaction in the context of infection identified an unappreciated role for epigenetics in the evolution of virulence in *S*. *aureus* and ultimately antimicrobial resistance in patients.

Recent reports link the *E*. *coli* prophage Φstx104-encoded methyltransferase (M.EcoGIII) to global transcriptional changes in a hypervirulent strain O101:H4 variant that is associated with an outbreak of hemolytic uremic syndrome (26). Thus, the phenomenon of prophage methyltransferase acquisition in epidemic strains may be common. However, the contribution of M.EcoGIII transcriptional regulation to virulence of *E*. *coli* O101:H4 is confounded by the observation that ΦStx104 encodes Shiga toxin genes *stxA* and *B* that cause hemolytic uremic syndrome. To our knowledge, mΦ11 is the only example in which differential regulation of a core virulence factor by a phage-encoded methyltransferase is sufficient for elevated virulence during infection.

To date, studies on the effect of prophages in pathogenesis focus largely on the role of prophage-encoded secreted toxins and immune modulators (51). Nevertheless, many, if not most, prophages lack known virulence or fitness factors (12) and frequently contain orphan methyltransferases (52). Thus, our findings, and those in *E*. *coli*, support the idea that prophage-mediated regulation of host bacterial gene expression may be a critical feature of the bacterium-phage interaction. In this connection, we note that ΦN315 phage-encoded *sprD*, a regulatory RNA, can enhance virulence in *S*. *aureus* (53, 54). This finding suggests the existence of alternative routes to hypervirulence with phage that involves gene regulation.

Our results, and those of M.EcoGIII in *E*. *coli*, also support the idea that DNA methylation has implications beyond that of bacterial defense against foreign DNA. Recent work showed that horizontal acquisition of phage-encoded methyltransferases and associated changes in gene expression are linked to speciation in *V*. *cholera* (55) and *M*. *abscessus* (56). In contrast, although restriction-modification systems are well-characterized in *S*. *aureus* (reviewed in ref. 57), little is known about the role of methyltransferases in staphylococcal gene regulation. Thus, the present study forms a knowledge base whereupon the role of methyltransferase in the pathogenesis of staphylococcal disease can now be interrogated by genome-wide mapping to assess the role of DNA modification events in staphylococcal virulence.

Three additional comments are relevant to the work described above. First, our present and prior (5) findings that mΦ11 phenotypes are specific to in vivo analyses supports the notion that in vitro analyses of virulence genes and regulators do not necessarily correlate with virulence during infection (58). This may be especially true for phage-mediated virulence, where induction is often critical to stimulate expression of phage-encoded genes (59). Therefore, future investigations to determine prophage effects on virulence should prioritize in vivo models. Second, the observation that biofilm is associated with virulence during skin abscesses caused by USA300 is concordant with a prior study linking biofilm and deep tissue abscesses (41). Accordingly, we suggest that future investigation of MRSA skin infection should consider that there may be similar pathogenesis mechanisms to infectious syndromes that are traditionally associated with MRSA biofilm (e.g., device infections, endocarditis, osteomyelitis). Finally, we demonstrated that *fnbA* was critical in the *pamA* virulence pathway, and our finding that skin infection virulence was increased without affecting dermonecrosis is consistent with previous findings on *S*. *aureus* surface-associated proteins including FnBPA (41). We found that the *pamA-fnbA* virulence pathway acts by increasing biofilm production, but we recognize that FnBPA may have additional functions that promote skin infection and abscess formation (32, 39, 60) that were not examined in this study. Furthermore, although our proteomic and transcriptomic analyses did not identify other biofilm-associated genes significantly altered by *pamA*, our study does not definitively rule out the possibility that other biofilm-related genes may be contributing to the increased biofilm phenotype. Future work will examine these mechanisms in the *pamA* context.

In summary, our study identifies molecular mechanisms underlying the relationship between prophage, virulence, and the emergence of an adapted CA-MRSA clone. Several questions concerning the role of phage-encoded methyltransferases are raised by the present study. For example, what is the host tissue-specific signal in vivo and does it require phage (or phage gene) induction? Moreover, how does *pamA* alter methylation patterns and what specific difference in methylation is responsible for increased *fnbA* expression and the enlarged abscess phenotype? Third, is the phage-encoded methyltransferase mechanism of gene regulation useful in other ecological contexts, such as colonization? Last, what is the frequency and distribution of orphan methyltransferase in natural populations of phage and *S*. *aureus*? Implicit in these questions is the view that phages are independently evolving entities that have acquired pathogenesis-adaptive genes. This behavior optimizes their existence as parasites, which they have fine-tuned through multiple functions that enhance the fitness of their bacterial hosts.

## Methods

### Sex as a biological variable.

Initial experiments comparing LAC* and LAC*/ mΦ11, which established the mΦ11 skin abscess virulence phenotype, were performed in both male and female mice and showed a similar increased skin abscess size phenotype but less variability in the female cohort (data not shown). Because of the reduced variability in female animals, experiments in the current study exclusively examined female mice. We believe that the findings are likely relevant to both sexes, but based on our current data, it is unknown whether the findings are relevant for male mice.

### Bacterial strains and growth conditions.

Bacterial strains, plasmids, and oligonucleotides used in this study are described in Supplemental Tables 2 and 3. *S*. *aureus* colony formation was on 5% sheep blood agar or tryptic soy agar (TSA) plates, and *E*. *coli* was on Luria Bertani plates. *S*. *aureus* strains were grown in tryptic soy broth (TSB) medium at 37°C with orbital shaking at 4*g*. Plates and media were supplemented with selective antibiotics when appropriate (ampicillin 100 μg/mL, chloramphenicol 10 μg/mL, erythromycin 5 μg/mL, or CdCl_2_ 0.4 mM, unless stated otherwise). Transductions were performed with phage 80α using established protocols (61); transductants were selected on TSA plates with appropriate antibiotics. PCR amplifications used Phusion Plus PCR Master Mix (Thermo Fisher Scientific, F631) and oligonucleotides from Integrated DNA Technologies (IDT). Detailed strain construction methods are provided in Supplemental Methods section A. Briefly, strains containing in-frame deletions (RU39, RU42, RU47, and RU108) were engineered in strain LAC*/mΦ11 (BS989) by allelic exchange with cloning plasmid pIMAY (62). Deletions were confirmed by Sanger sequencing (Psomagen, Inc.) and comparative sequence analysis (Supplemental Figures 2 and 3), as outlined below.

Strains containing a single-copy chromosomal insertion of a constitutively expressed *pamA* (P*_sarA_*-*sod*RBS-*pamA*) or empty vector were generated by insertion of pRU7 or pJC1111, respectively, at the *S*. *aureus* pathogenicity island 1 (SapI1) site of strain BS656 (27), and then by transducing the mutation with phage 80α into LAC* (resulting in strains RU121 and RU131), LAC*/mΦ11Δ*pamA* (resulting in strains RU129 and RU128), or BVED028 (resulting in strains RU241 and RU242). The presence and location of the P*_sarA_*-*sod*RBS-*pamA* and empty vector inserts were confirmed by PCR.

To construct strains with inactivating *pamA* point mutations, complementary oligonucleotides that contained the desired *pamA* mutation were used to amplify P*_sarA_*-*sod*RBS-*pamA* from pRU7 template DNA, and then the amplification products were sewn together by overlap extension PCR (63). P*_sarA_*-*sod*RBS-*pamA*P65A, P*_sarA_*-*sod*RBS-*pamA*P65T, and P*_sarA_*-*sod*RBS-*pamA*P66A fragments were inserted into pJC1111 with Gibson assembly, resulting in pRU8, pRU9, and pRU10, respectively. pRU8, pRU9, and pRU10 were transformed into competent *E*. *coli* DH5α (New England Biolabs, C2987H) per the manufacturer’s instructions, electroporated into BS656 for insertion into the SapI1 *att* site (27), and transduced into LAC* with phage 80α, resulting in strains RU161, RU162, and RU164, respectively. Point mutations were confirmed by Sanger sequencing.

To construct strains with *fnbA:bursa* transposon insertions, phage 80α lysate of strain NE186 (*fnbA*:*bursa*, Erm^r^) (64) was used to transduce RU121, RU129, and BS989, generating RU169, RU170, and RU171, respectively. PCR amplification across the *fnbA:bursa* insertion site was performed to confirm the transposon insertion.

### Mapping deletions using whole genome sequencing.

Extracted purified gDNA was quantified with the Quant-iT PicoGreen dsDNA assay kit (Invitrogen, P7589) prior to library prep. Samples were normalized by concentration and libraries prepared with the Illumina DNA prep (M) Tagmentation kit (catalog 20018705). Each library was combined equimolar and sequenced as paired-end 150 bp reads using the Illumina NovaSeq 6000 system with the S1 300 cartridge and flow cell.

For whole genome sequencing analysis, BWA v0.7.17 (65) was used to map the raw short-read sequences of the *S*. *aureus* samples (strains LAC*, BS989, RU39, RU42, RU47, and RU108) to an *S*. *aureus* reference assembly (NCBI accession GCF_015475575.1); https://www.ncbi.nlm.nih.gov/datasets/genome/GCF_015475575.1/) and the mΦ11 phage (GenBank accession PP554657), resulting in 1 alignment file per sample. The depth of mapped reads was computed with bedtools v2.30.0 (66) using the command bedtools genomecov -iBAM file.bam -d, where file.bam stands for each of the sample alignment files. This procedure generated output files tabulating contig name, start site, end site, and number of reads covering each base. Read coverage per base were loaded into R v4.2.0 for visualization. Plots were created with ggplot2 (67).

### Growth curves.

Overnight cultures were diluted (1:1,000) into fresh TSB or RPMI medium (Sigma-Aldrich, R6504), and growth was monitored at 37°C in 100-well (150 μL well) honeycomb plates (Thermo Fisher Scientific, 12871511), using a Bioscreen C Analyzer (Thermo Labsystems), measuring OD_600_ at 30-minute intervals. The curves represent averaged values from 3 biological samples. Each biological sample was run as 10 technical replicates.

### Secreted protein preparation.

Overnight cultures were pelleted, washed with sterile PBS, OD normalized, and diluted 1:200 into TSB medium for growth at 37°C with shaking at 4*g*. After 6 and 24 hours, cells were centrifuged at approximately 3,200*g* for 15 minutes to remove bacteria, and aliquots (1.3 mL) of supernatant were passed through a 0.2 μM filter (Thermo Fisher Scientific, 723-2520). The supernatants were precipitated with 100% trichloroacetic acid using established protocols (68).

### Animal infections.

Five-week-old female Swiss Webster mice (Envigo/Inotiv) were anesthetized with Avertin (2,2,2-tribromoethanol dissolved in *tert*-Amyl alcohol and diluted in sterile PBS to a final concentration of 2.5% vol/vol) via intraperitoneal injection (300 μL). Mice were shaved with mechanical clippers, and approximately 1 × 10^7^ CFU of bacteria was injected (100 μL) subcutaneously into each flank using Adson forceps (69). For daily abscess measurements, mice were briefly anesthetized with inhaled isoflurane. Abscess diameter was measured with digital calipers (Thermo Fisher Scientific, 14-648-17). Abscess area and dermonecrosis area were quantified using digital photography and ImageJ (NIH) (70). Measurements were standardized to a centimeter ruler in frame. At 72 hours after infection, mice were euthanized and abscesses excised with an 8 mm punch biopsy (Integra Life Sciences). Tissue biopsies were either prepared for histology as described below or homogenized for CFU enumeration or cytokine analysis. If both CFU enumeration and histology or other preparation was required, left flank biopsies were homogenized for CFU enumeration while right flank biopsies were used for the additional analysis, to minimize bias. For homogenization, biopsy samples were added to 2 mL conical screw cap tubes (Thermo Fisher Scientific, 023-681-344 and 02-681-358) with sterile PBS (1 mL) and a single 0.25” ceramic sphere (MP Biomedicals, 116540034), weighed, and homogenized by 3 cycles in a FastPrep-24 homogenizer (MP Biomedicals) at 4 m/s for 60 seconds. Homogenates were serially diluted in sterile PBS and plated on TSA for CFU enumeration. For cytokine analysis, 1× Halt protease inhibitor cocktail (Thermo Fisher Scientific, 78429) was added to homogenates, and samples were stored at –80°C.

### Histology.

Skin biopsies were immobilized in cassettes (Simport Scientific, M490-2), fixed in 10% formalin for 72 hours at 4°C, washed in sterile PBS 3 times for 20 minutes, and then dehydrated with increasing concentrations of ethyl alcohol (EtOH) before storage in 70% EtOH at 4°C. Fixed and dehydrated specimens were embedded in paraffin, and 5 μm sections were made through the center of the abscess for H&E and Gram staining. Slides were scored for inflammatory burden (mild/moderate/severe) and abscess architecture (nodular/diffuse) by a board-certified dermatopathologist who was masked to the sample identity throughout.

### Cytokine analysis.

Skin abscess cytokine profiles were obtained using the MILLIPLEX MAP Mouse Cytokine/Chemokine Magnetic Bead Panel (MilliporeSigma, MCYTMAG-70K-PX32). Samples were prepared per the manufacturer’s instructions. Data were acquired using a Luminex MABPIX instrument and analyzed using xPONENT software (MilliporeSigma). Statistical analyses were performed for each individual cytokine.

### DpnI digestion.

gDNA was extracted from strains RU121, RU129, RU161, RU162, and RU164 for Figure 4B and BS819, BS989, BS990, RU39, RU121, and RU129 for Supplemental Figure 7; digested using DpnI (New England Biolabs, R0176S) per the manufacturer’s protocol; separated on a 1% agarose gel containing SYBR Safe (Thermo Fisher Scientific, S33102); and imaged in a ChemiDoc imager (Bio-Rad).

### RNA preparation and sequencing.

For transcriptional profiling of strains LAC* and LAC*/mΦ11 (Supplemental Figure 1F), 2 independent overnight cultures were diluted (1:100) into fresh TSB medium (5 mL) and grown at 37°C shaking at 4*g* to early (3 hours) or late exponential growth phase (6 hours). For transcriptional profiling of strains LAC*:EV and LAC*:*pamA* (Figure 5, A and B), 3 independent overnight cultures were diluted (1:100) into RPMI medium (15 mL) and incubated at 37°C with shaking at 4*g* to exponential growth phase (5 hours).

For RNA extraction, cells were concentrated by centrifugation (3,400*g* for 5 minutes), resuspended in 1 mL TRIzol (Invitrogen, 15596026), and disrupted using lysis matrix B (MP Biomedicals, 116911050) tubes in a FastPrep-24 (MP Bio) at 6 m/s, for 30 seconds, 3 times. Samples were centrifuged at 12,000*g* for 10 minutes at 4°C, and the upper phase was transferred into a new RNA-free tube containing ice cold TRIzol (500 μL), gently mixed, and incubated (5 minutes) at room temperature. Then chloroform (200 μL) was added, and samples were centrifuged at 12,000*g* for 15 minutes at 4°C. The aqueous phase was mixed with isopropanol (500 μL) and transferred to RNeasy column (QIAGEN, 74004) for washing and RNA elution. RNA was visualized on the Agilent 2100 Bioanalyzer system using a Bioanalyzer Nanochip run with the Prokaryote setting. Libraries were prepared with total RNA (500 ng per sample) of the high-quality samples (RNA integrity number 9–10) using the Illumina stranded Total RNA Prep Ligation with Ribo-Zero Plus kit (catalog 20040529) per the manufacturer’s instructions. PCR amplification was run with 11 total cycles. The libraries were visualized on the Agilent 4200 TapeStation System, and concentration was quantified by Qubit (Thermo Fisher Scientific). Libraries were pooled equimolar and sequenced as paired-end 50 bases on the Illumina NovaSeq 6000 system on 1 lane of the Illumina SP 100 cycle flow cell kit.

### RNA-Seq analysis.

For RNA-Seq of strains LAC* and LAC*/mΦ11 (Supplemental Figure 1F), we used previously established analysis methods (71), with the addition of the mΦ11 sequence to the reference genome. For RNA-Seq of strains LAC*:EV and LAC*:*pamA* (Figure 5A), we created a reference assembly by appending the pJC1111 sequence to the AH-LAC assembly (NCBI accession GCF_015475575.1) and annotating with NCBI’s Prokaryotic Genome Annotation Pipeline (PGAP) (72). However, the sequence of *pamA* was too short for processing by PGAP, so we manually added it to the gff and GenBank annotation files that PGAP produced. We used Bowtie2 v2.4.1 (73) to align the raw short-read sequences of LAC*:*pamA* and LAC*:EV to the reference assembly. Using the alignment files generated for each sample, the *featureCounts* command in Subread v2.0.1 (74) was used to count the reads mapping to each gene in the reference. Read counts per gene and sample were loaded into R v4.2.0 for differential expression analysis using the package DESeq2 v1.36.0 (75). The function DESeq with default settings was used to normalize for library size differences, to estimate dispersion, and to fit negative binomial generalized least squares models for each gene. Differential expression testing was performed using the Wald test as implemented by DESeq2. The resulting *P* values were adjusted using an FDR of 10%.

### qRT-PCR.

RNA was isolated from LAC*:*pamA* and LAC*:EV at exponential growth as described above. DNA was removed with Turbo DNase DNA free kit (Invitrogen, AM2238), and cDNA was synthesized using the Superscript III First-Strand Synthesis System (Invitrogen, 18080051). qRT-PCR was performed using TaqMan Universal PCR Master Mix (Thermo Fisher Scientific, 4304437) and primers/probes (IDT) specific to *pamA*, *fnbA*, and *rpoB* (Supplemental Table 3). Three independent biological samples of each strain were run in duplicate, and *rpoB* was used to normalize gene expression. Settings on the C1000 CFX96 machine (Bio-Rad) were as follows: 50°C for 2 minutes, 95°C for 10 minutes, and then 40 cycles (95°C for 15 seconds and 60°C for 1 minute). The 2^–ΔΔCt^ method was used to calculate the relative fold gene expression (76).

### In vitro biofilm assays.

Overnight broth cultures were diluted (1:100) into fresh TSB medium supplemented with 0.25% glucose (TSBG), aliquoted into 96-well (200 μL-well) tissue culture–treated polystyrene plates (Corning, CLS3799), and incubated statically at 37°C for 24 hours. Supernatants were discarded, and adherent biofilms were washed 3 times with sterile PBS (200 μL). For crystal violet biofilm quantification (Figure 6, A and F), washed biofilms were fixed with 100% ethanol (200 μL) and stained with crystal violet 0.1% w/v (200 μL) at room temperature for 15 minutes. Residual stain was discarded and biofilms were washed 3 times. Crystal violet was eluted with 33% acetic acid (200 μL), incubated for 10 minutes, and then samples were diluted (1:4) in PBS and quantified by measuring OD_595_ using a Synergy Neo2 plate reader (BioTek). For biofilm extracellular DNA (eDNA) quantification (Supplemental Figure 8B), overnight cultures were diluted (1:100) into fresh TSBG, aliquoted into 6-well (1.5 mL) tissue culture–treated polystyrene plates (Corning, CLS3516), and incubated statically at 37°C for 24 hours. Resuspension, filtration, and eDNA measurement using SYTOX Green (Thermo Fisher Scientific, S7020) was performed as previously described (77).

### Biofilm scanning electron microscopy.

Clean, 12 mm, glass coverslips (Thermo Fisher Scientific, 50-192-9516) were placed inside each well of a 6-well tissue culture–treated sterile dish (Corning, CLS3516) and incubated overnight at 37°C with sterile TSBG. After incubation, the medium was discarded, and overnight broth cultures of the indicated strains were diluted 1:100 into TSBG; then 3.6 mL was added to each well and incubated statically at 37°C for 24 hours. The wells were washed with sterile PBS 3 times, and then biofilms were fixed in 2.5% glutaraldehyde in PBS (pH 7.2) at room temperature for 1 hour, washed 3 times with PBS, postfixed with 1% osmium tetroxide (OsO_4_) in aqueous solution for 1 hour, and then dehydrated in a series of ethanol solution (30, 50, 70, 85, 95, 10 minutes each at room temperature) and finally with 100% ethanol 3 times for 20 minutes each. Glass coverslips with biofilm were critical point–dried using Tousimis Autosamdri-931 critical point dryer, mounted on scanning electron microscopy stubs covered with double-sided electron-conducted tape, coated with gold/palladium by Safematic CCU-010 SEM coating system (Rave Scientific), and imaged by Zeiss Gemini300 FESEM using secondary electron detector (SE_2_) at 5 kV with working distance from 5.4 to 8.1 mm.

### Biofilm cell wall–associated protein preparation.

Overnight cultures were diluted (1:100) into fresh TSBG, aliquoted into 6-well (3.6 mL) tissue culture–treated polystyrene plates (Corning, CLS3516), and incubated statically at 37°C for 24 hours. Supernatants were discarded, and biofilms were washed 2 times with 3.6 mL sterile PBS, resuspended in 1 mL sterile PBS, normalized to OD_600_, and centrifuged (12,000*g* for 2 minutes). Biofilm pellets were washed twice with PBS (1 mL), resuspended in a 48 μL mixture of lysostaphin (20 μg/mL), 1× Halt protease inhibitor in TSM buffer (10 mM MgCl_2_ and 500 mM sucrose in 50 mM Tris, pH 7.5), and incubated for 30 minutes at 37°C. Samples were centrifuged (12,000*g* for 2 minutes), and the supernatant (36 μL) was mixed with 4× SDS (12 μL) sample buffer (200 mM Tris-HCl, pH 6.8; 588 mM β-mercaptoethanol; 8% SDS; 0.08% bromophenol blue; 40% glycerol, 50 mM EDTA). Samples were boiled for 10 minutes and stored at –80°C.

### Coomassie staining and immunoblotting.

Proteins were separated by SDS-PAGE (12% gel; Bio-Rad, 4561043), visualized using InstantBlue Coomassie dye (Abcam, 50-196-3787), and transferred to nitrocellulose membrane for analysis by immunoblot. The membrane was incubated in Everyblot blocking buffer (Bio-Rad, 12010020) for 5 minutes of blocking at room temperature, then primary antibody (1:2,000 dilution of anti-FnBPA antibody, Abnova, PAB16068) overnight at 4°C, and then secondary antibody (1:25,000 dilution of Alexa Fluor 680–conjugated goat anti-rabbit IgG, Invitrogen, A21076) for 1 hour at room temperature. Images were acquired with the Odyssey Clx imaging system and Image Studio software (Li-Cor Biosciences).

### Protein identification by mass spectrometry.

High–molecular weight bands noted on the LAC*:*pamA* biofilm cell wall–associated protein preparation (Figure 6D) were manually excised from gel lanes and stored in 1 mL 1% acetic acid. As a control, similar high–molecular weight areas in LAC*:EV lanes and 1 PBS control lane were excised in the same manner. The proteins were in-gel digested using trypsin as previously described (78). Sample processing, mass spectrometry, and data analysis were performed as described in Supplemental Methods section B.

### Quantification of abscess tissue biofilm by immunohistochemistry.

The following methods were adapted from prior work (48). Skin biopsies were fixed with periodate-lysine-paraformaldehyde buffer overnight at 4°C, dehydrated in sucrose (30%) for 24 hours, and frozen in OCT compound (Thermo Fisher Scientific, 1437365). For *S*. *aureus* staining, 10 μm–thick skin sections were incubated in BSA (2%) in Tris-buffered saline (TBS) with the primary antibody (1:400 dilution of rabbit anti–*S*. *aureus*; Abcam, 20920) at 4°C overnight. The sections were washed 3 times with 1% BSA in TBS and incubated with the secondary antibody (1:500 dilution of goat anti-rabbit IgG-AF488; Invitrogen, A-1108) and DAPI at 4°C for 1 hour. For 5-mC staining, 10 μm–thick skin sections were permeabilized with hydrogen chloride (1.5 M, Fisher Chemical) to allow the 5-mC antibody to stain the biofilm. The sections were washed twice with PBS and incubated in 2% BSA plus TBS with primary antibody (1:80 dilution rabbit anti–5-mC antibody; Cell Signaling Technology, D3S2Z) at 4°C overnight. The sections were washed 3 times with 1% BSA in TBS and incubated with secondary antibody (1:500 dilution goat anti-rabbit IgG-AF488; Invitrogen, A-1108) and DAPI at 4°C for 1 hour. The sections were again washed 3 times with 1% BSA in TBS and then mounted with cover glass over tissue sections using ProLong Diamond Antifade Mountant (Invitrogen). All the antibodies were diluted in blocking solution. Imaging and analysis were performed as previously described (48), with the exception that thresholds of 5-mC and DAPI fluorescence intensity were determined based on the staining from mock-infected (sterile PBS) skin biopsies.

### Statistics.

For comparisons of 2 groups, normality was determined using the Shapiro-Wilk test. If data were normally distributed, 2-tailed unpaired *t* tests were performed. If data were not normally distributed, a Mann-Whitney test was used. For comparison of more than 2 groups, if normality was determined, 1-way ANOVA with Tukey’s multiple-comparison test was used. If any group was not normally distributed, a Kruskal-Wallis test with multiple comparisons was performed. A *P* value less than or equal to 0.05 was considered significant. Analyses were performed using GraphPad Prism version 9.4.1 for Macintosh.

### Study approval.

All animal experiments were approved by the IACUC (protocol 107203) of NYU Langone Medical Center. All experiments were performed according to *Guide for the Care and Use of Laboratory Animals* (National Academies Press, 2011) and US federal law.

### Data availability.

Values for all individual data points are reported in the Supporting Data Values file. RNA-Seq files are deposited in NCBI’s Gene Expression Omnibus (GEO GSE25535, corresponding to Supplemental Figure 1F, and GSE252862, corresponding to Figure 5A). Whole genome sequencing of *S*. *aureus* strains constructed during this study is deposited in the NCBI’s Sequence Read Archive (SRA BioProject PRJNA1090089). The sequence of prophage mΦ11 can be accessed as GenBank accession PP554657. Additional data will be made available upon request.

## Author contributions

RJU, BS, and VJT designed the study; KD provided contributions to the conception and design of the work; RJU, MP, RT, II, KAL, DB, SMM, SM, TKK, EEZ, CZ, and RHK performed experiments and generated data; RJU, RT, SM, GP, NMS, AP, and HVB analyzed data; RJU, BS, and VJT secured funding; RJU wrote the initial manuscript draft. All authors contributed substantial revisions and approved the final version of the manuscript.

## Supplementary Material

Supplemental data

Unedited blot and gel images

Supporting data values

## Version 1 07/23/2025

In-Press Preview

## Version 2 09/16/2025

Electronic publication
