# Supplementary material for: Prophage-encoded methyltransferase drives adaptation of community-acquired methicillin-resistant Staphylococcus aureus
Source: J Clin Invest. 2025 Jul 22;135(18):e177872. doi: 10.1172/JCI177872 (PMC12435837; doi:10.1172/JCI177872)
Supplement: Supplemental data [file jci-135-177872-s057.pdf]

Supplemental Figures and Tables

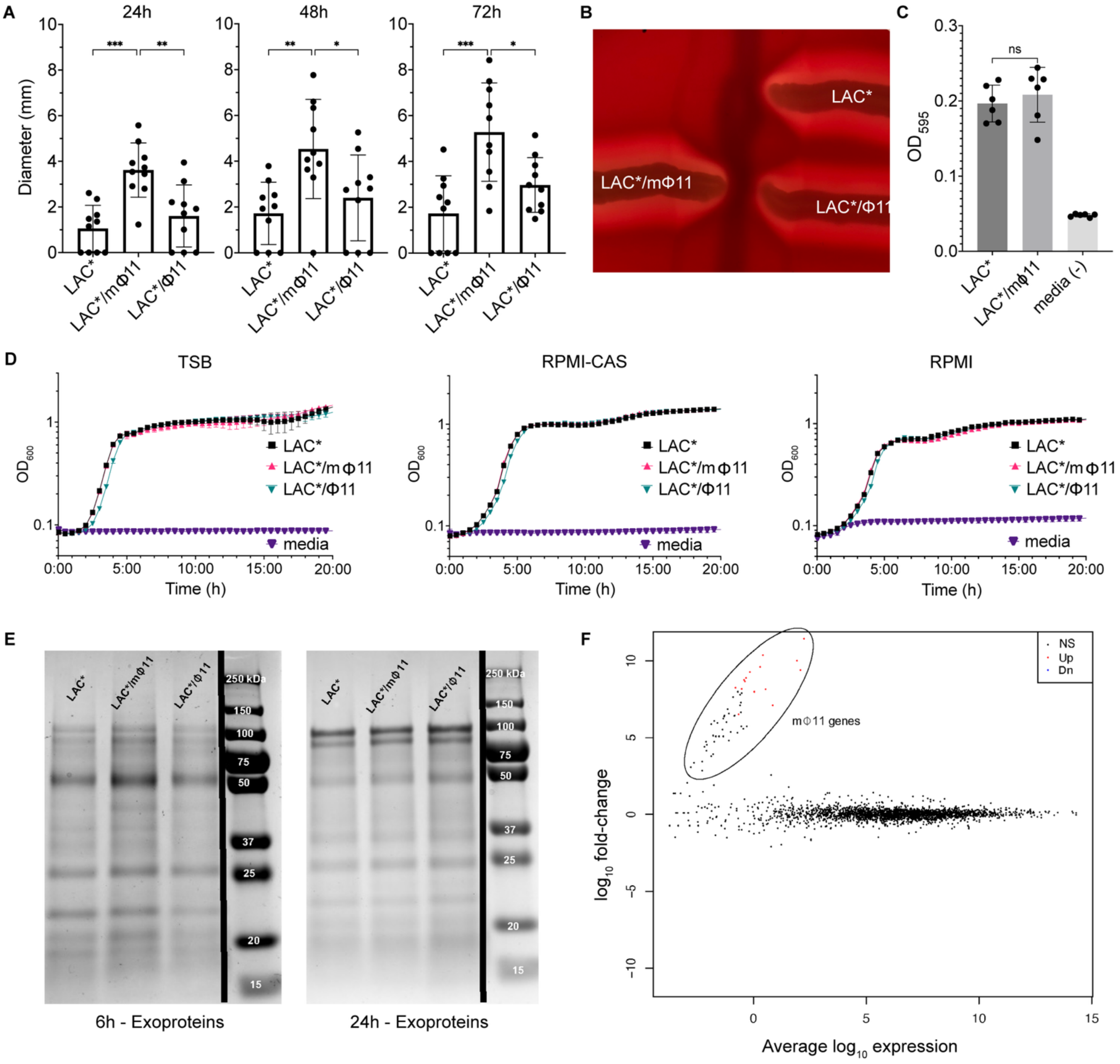

Supplemental Figure 1. mΦ11 increases skin abscess size but does not affect in vitro growth, hemolysis, biofilm production, exoproteins, or transcription of non-phage genes. **(A)** Skin abscess diameter (N=10 abscesses per strain) of LAC*, LAC*/mΦ11 (strain BS989), and LAC*/Φ11 (strain BS990) at the indicated time points post-infection with ~10^7 bacterial CFU.

Data represent mean ± SD from one experiment that replicates prior findings (1). Statistical significance was determined by Kruskal-Wallis and Dunn's tests, *$P \leq .05$, **$P \leq .01$, ***$P \leq .001$. **(B)** Hemolysis patterns. The indicated strains were streaked perpendicular to β-hemolysin producing strain RN4220 (streaked vertically) on a sheep blood agar plate. Cross-streaking should differentiate *S. aureus* hemolytic activities by virtue of their synergism with β-hemolysin. However, hemolysis patterns did not change due to mΦ11. **(C)** In vitro biofilm production. Biofilms after 24 h of static incubation of LAC*, LAC*/mΦ11, or negative (media only) control were stained and quantified by OD . Data represent mean ± SD, pooled from two independent experiments. Statistical significance was determined by Mann-Whitney test. **(D)** Growth curves of the indicated strains in various media. Data are the mean ± SD of three biological replicates. Results represent two independent experiments. **(E)** Exoproteins were prepared from culture supernatants of the indicated strains after the indicated growth times and analyzed by SDS-PAGE. Black line indicates non-contiguous lanes. Images represent two independent experiments. **(F)** Scatter plot comparing log RNA transcript levels at late exponential (6 h) growth of LAC* vs LAC*/mΦ11 strains. Orange data points represent genes with increased expression in LAC*/mΦ11 meeting statistical significance ($P \leq .05$), which all fall within the circled area representing mΦ11 genes. Data is representative of two separate experiments.

Collectively, these data show that in vitro analyses do not correlate with phage content, suggesting that the mΦ11-mediated enhanced virulence in the skin requires host tissue-specific signals in vivo.

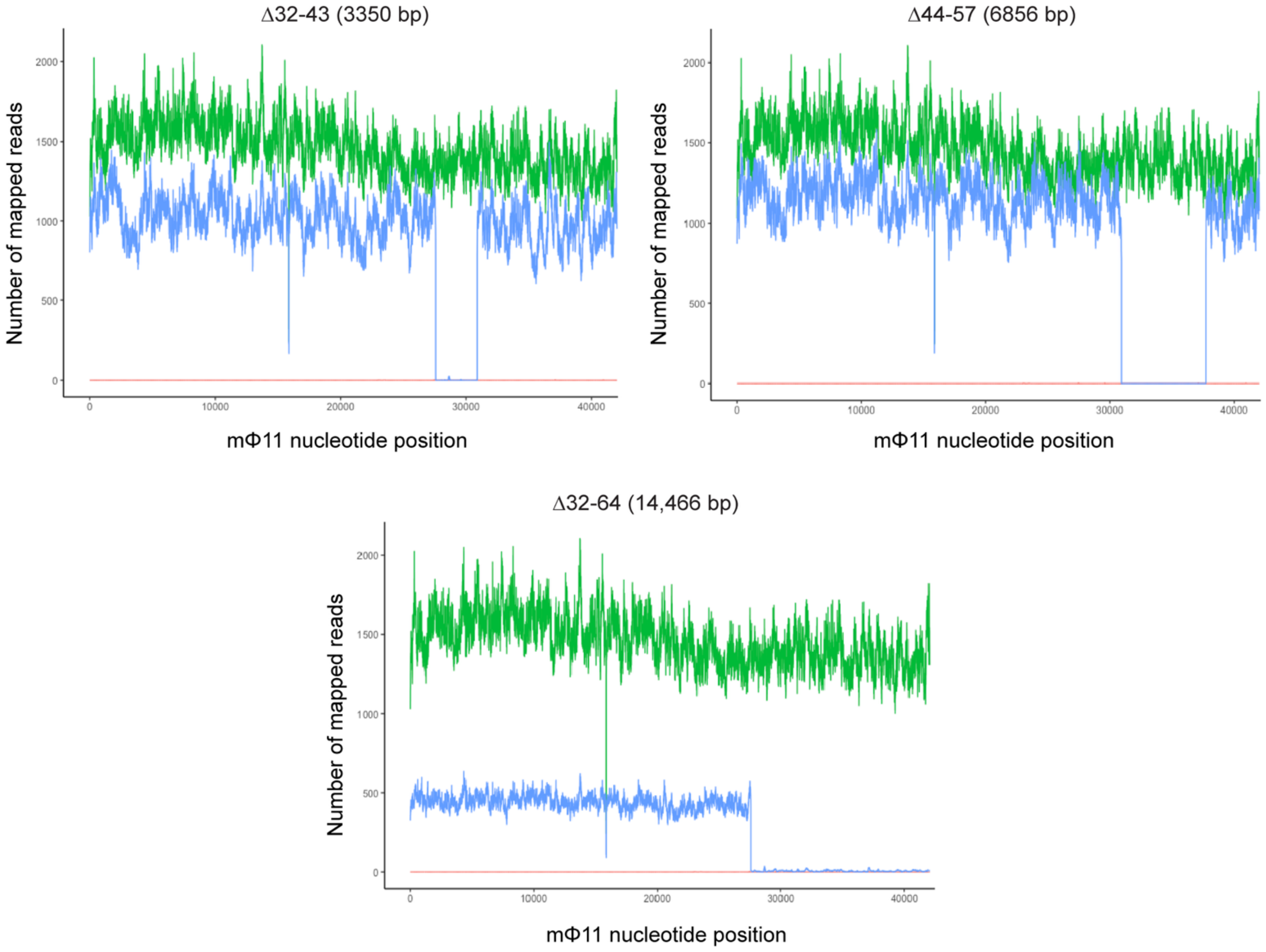

Supplemental Figure 2. Validation of en bloc deletion clones by whole genome sequencing. Genomic comparison of LAC*/mΦ11 (green, strain BS989), LAC*/mΦ11 containing the indicated en bloc deletion (blue, strains RU42, RU47 and RU108), and control LAC* (red) strains using bedtools v2.30.0 (2) with the mΦ11 sequence as a reference. The sharp cutoffs to zero mapped reads in the en bloc deletion strains correspond to the expected deletions in each strain.

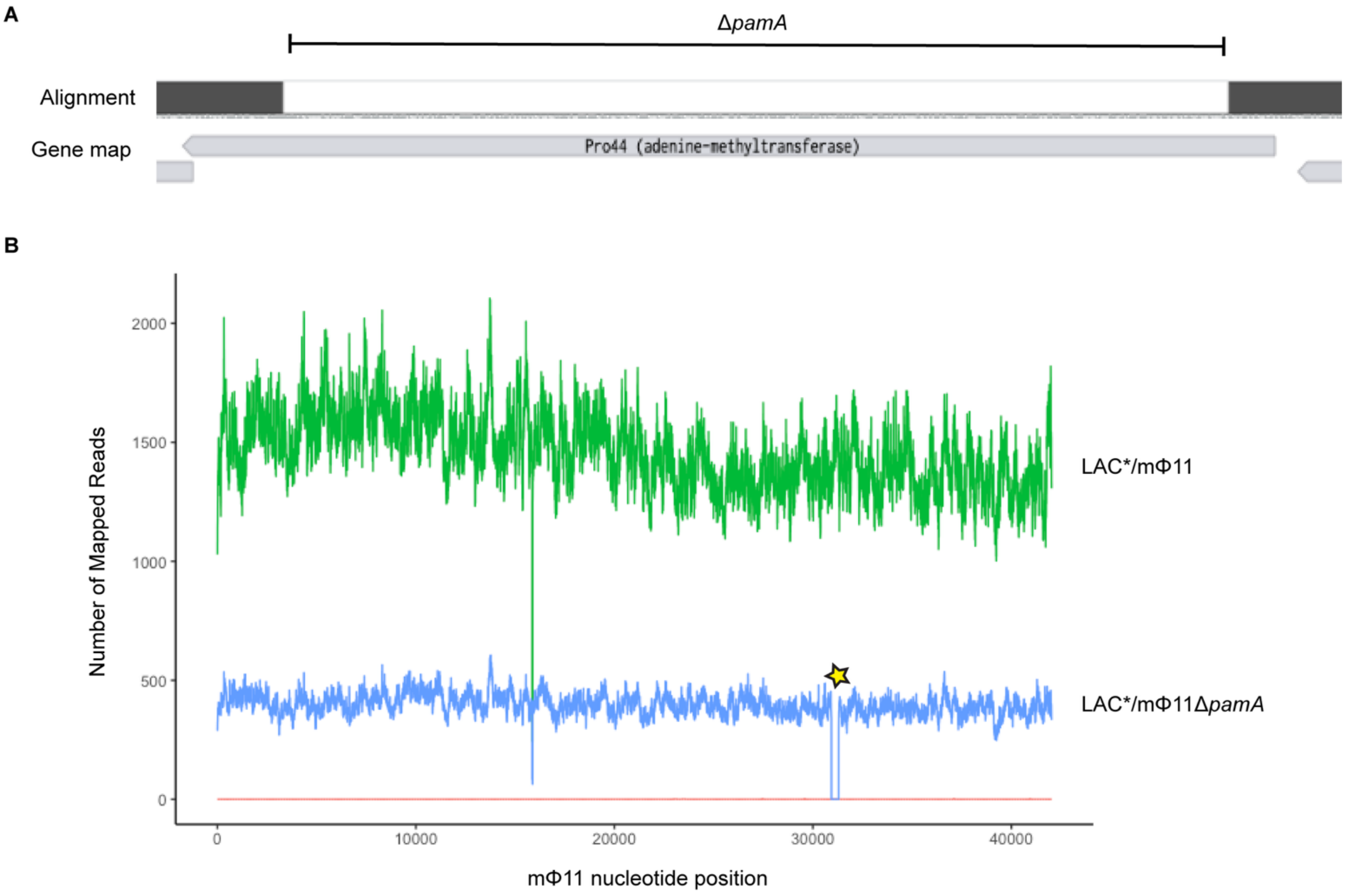

Supplemental Figure 3. Validation of unmarked, in-frame deletion of *pamA* by Sanger and whole genome sequencing. (A) Alignment of *pamA* sequence from LAC*/mΦ11Δ*pamA* (strain RU39) obtained using Sanger Sequencing to reference mΦ11 using Benchling alignment tool (https://www.benchling.com/). Area highlighted by bracket, with no alignment to the reference gene map, confirms the 369 bp deletion in Δ*pamA*. **(B)** Whole genome sequencing comparison of LAC*/mΦ11 (green, strain BS989), LAC*/mΦ11Δ*pamA* (blue, strain RU39), and control LAC* (red) to reference mΦ11, performed as in **Supplemental Figure 2**. This alignment shows that all reads from LAC*/mϕ11 are present in LAC*/mΦ11Δ*pamA* except for a gap in coverage corresponding to the *pamA* deletion (highlighted by yellow star).

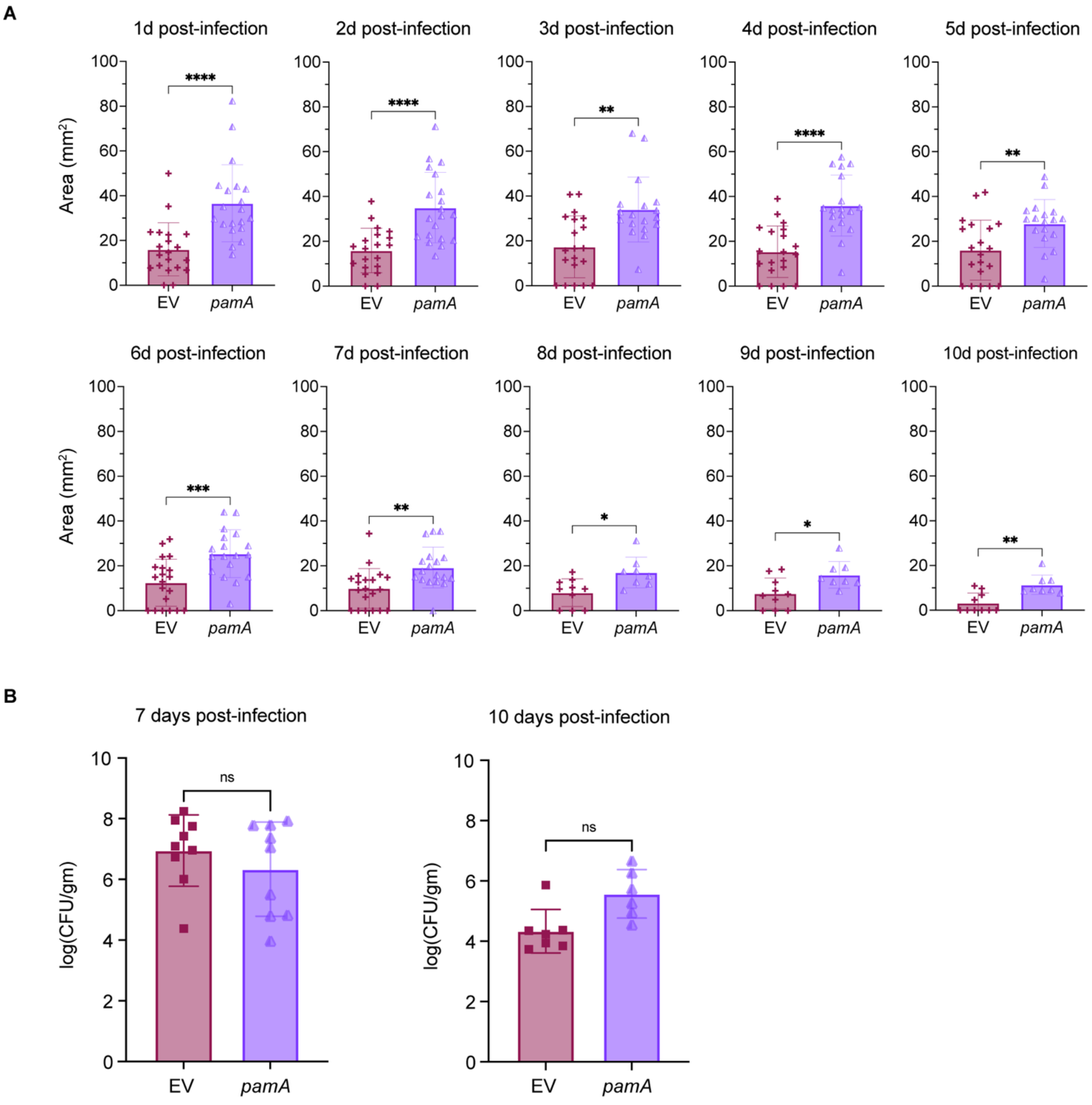

Supplemental Figure 4. *pamA* increases skin abscess size over an extended post-infection period without increasing bacterial burden. (A) Effect of *pamA* on skin abscess size over an extended (10 d) time course. Abscess area was measured after skin infection with ~1x10^7 bacterial CFU per abscess of LAC* with empty vector (EV) control (maroon, N=20

abscesses, strain RU129) or a single copy of constitutively expressed *pamA* (purple, N=20 abscesses, strain RU121) integrated into the chromosome. Data between 1-7 d post-infection are pooled from two independent experiments, and data between 8-10 d are from one experiment. These data represent mean ± SD, and significance was determined by Mann-Whitney test, **P*≤.05, ***P*≤.01, ****P*≤.001, *****P*≤.0001 **(B)** Effect of *pamA* on bacterial CFU recovered from skin abscesses. Left flank abscesses (N=7-10 abscesses per strain) from infections performed in panel A were harvested at 7 and 10 d post-infection. Data represent mean ± SD of CFU recovered, and significance was determined by Mann-Whitney test.

Collectively, these data show that the *pamA*-induced abscess size phenotype persists even as the abscesses resolve and, like our findings at the 72 h time point **(Figure 3B)**, the abscess size phenotype is independent of bacterial CFU recovery.

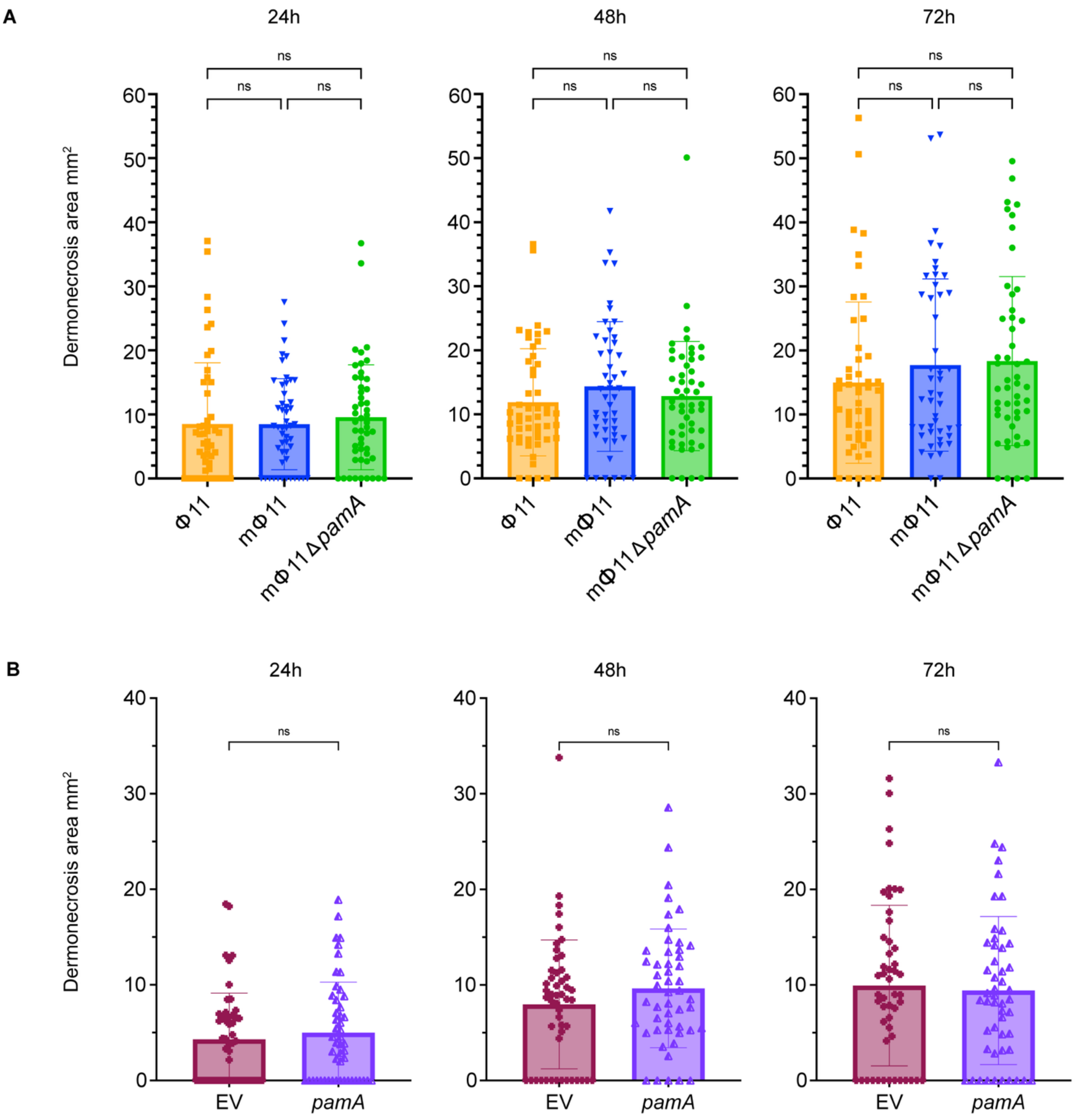

Supplemental Figure 5. *pamA* does not affect skin abscess dermonecrosis. (A) Effect of *pamA* on skin dermonecrosis in mΦ11 lysogens. Dermonecrosis area at the indicated time points post-infection with ~1.5x10^7 bacterial CFU of LAC* containing Φ11 (orange, N=50

abscesses, strain BS990), mΦ11 (blue, N=48-50 abscesses, strain BS989), or mΦ11Δ*pamA* (green, N=50 abscesses, strain RU39). Results are pooled from four independent experiments. Data represent mean ± SD. Statistical significance was determined by Kruskal-Wallis and Dunn's tests. **(B)** Effect of *pamA* on skin dermonecrosis without other mΦ11 genes. Dermonecrosis area of LAC* with empty vector (EV) (maroon, N=50 abscesses, strain RU129) or constitutively expressed *pamA* (purple, N=50 abscesses, strain RU121) integrated into the chromosome in single copy after infection in with ~1x10^7 CFU of bacteria per abscess for the indicated times. Data are pooled from four independent experiments and represent mean ± SD. Statistical significance was determined by Mann-Whitney test.

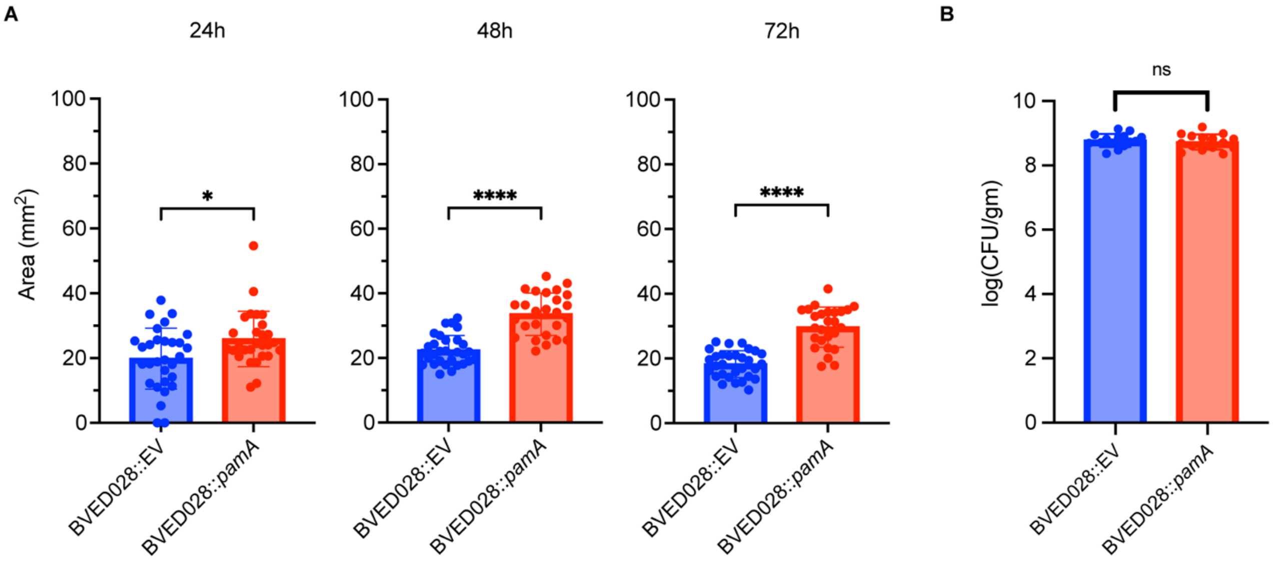

Supplemental Figure 6. *pamA* expression increases skin abscess size in MRSA isolate with clonal complex one (CC1) genetic background. (A) Skin abscess size. Skin abscess area of CC1 strain BVED028 with *pamA* expression vector (BVED028::*pamA*, N=30 abscesses) compared to empty vector control (BVED028::EV, N=30 abscesses) at the indicated time points after infection with ~$1x10^7$ bacterial CFU per abscess. Results are pooled from two independent experiments. Data represent mean ± SD. Statistical significance was determined by unpaired t-test, **P*≤.05, *****P*≤.0001. **(B)** Skin bacterial burden at 72 h post-infection. Skin abscesses (N=15 abscesses per strain) from the left flank of the infected animals included in panel A were harvested at 72 h post-infection and CFU enumerated. Data represent mean ± SD. Statistical significance was determined by unpaired t-test. Together, these data show that the *pamA* phenotype—increased abscess size independent of bacterial burden—can extend to strains outside of USA300 (CC8).

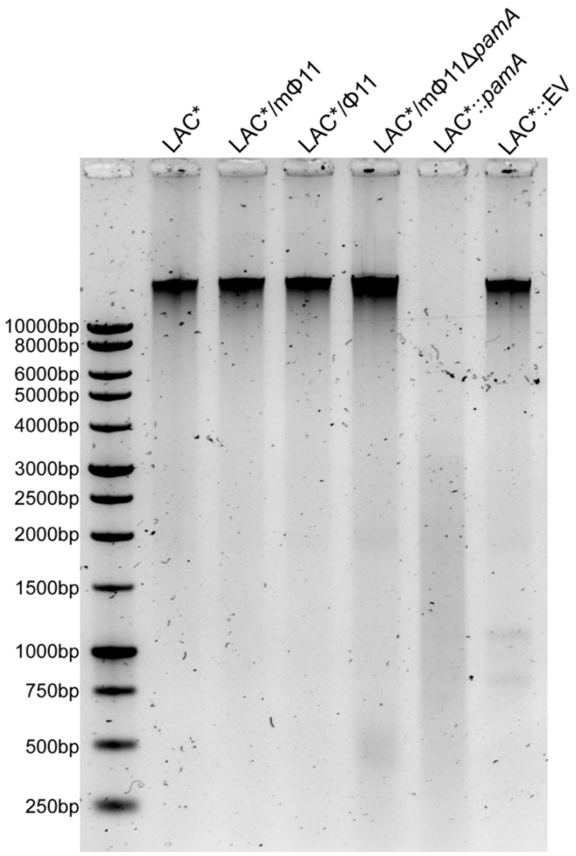

Supplemental Figure 7. DpnI digestion of parental and *pamA* expression vector strains after in vitro growth. Genomic DNA (gDNA) from LAC* containing the indicated alleles was isolated after overnight growth in tryptic soy broth, digested with DpnI (DpnI+), and visualized on 1% agarose gel. The complete digestion of gDNA from LAC*::*pamA,* together with the absence of digestion of the parental or empty vector (EV) control strains' DNA, shows that *pamA* requires a constitutive promoter to be expressed and induce gDNA methylation during in vitro growth. These data at least partially explain the lack of in vitro phenotypes in parental mΦ11 lysogens **(Supplemental Figure 1B-F)** despite *pamA*-induced phenotypes during infection **(Figures 2-3)**. The *pamA* expression vector in LAC*::*pamA* bypasses the need for in vivo signaling from the infectious environment, thereby allowing for in vitro exploration of *pamA*-related phenotypes **(Figures 5-6)**.

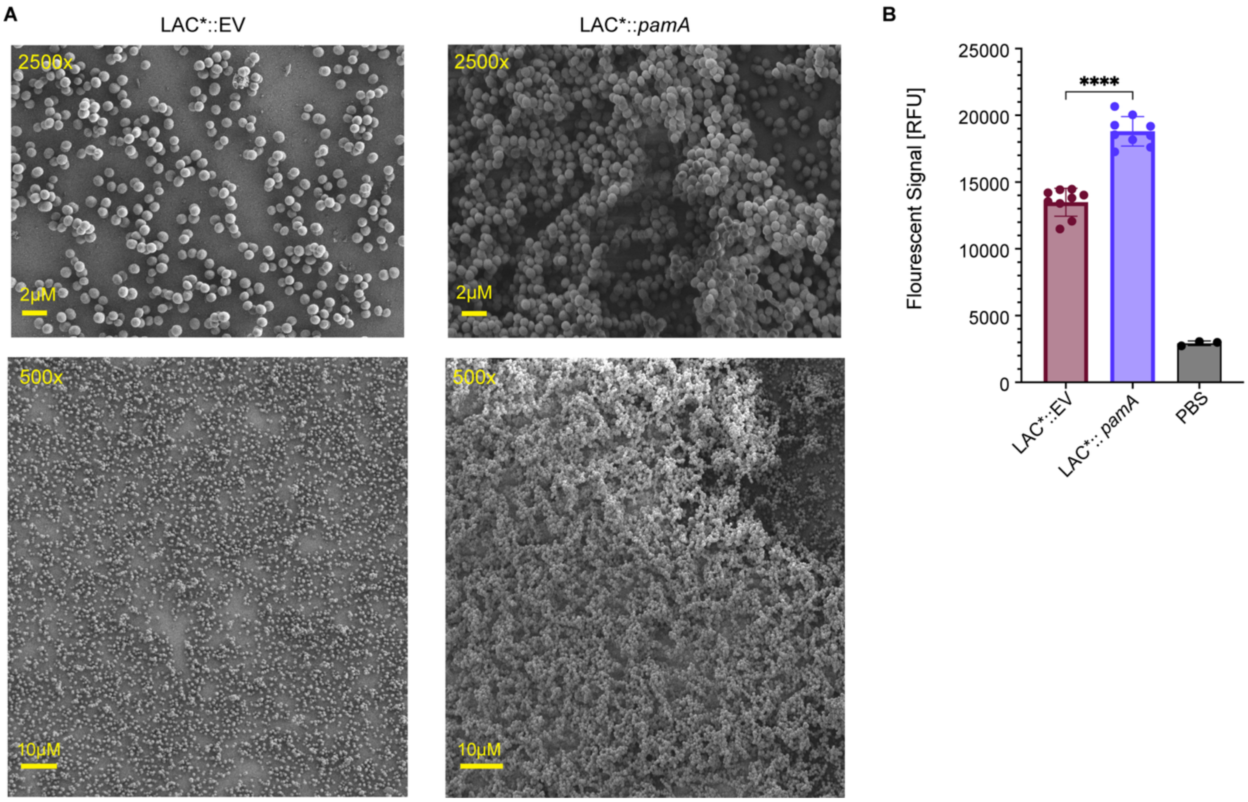

Supplemental Figure 8. *pamA* induces a robust in vitro biofilm architecture and increases biofilm extracellular DNA (eDNA). (A) Scanning electron microscopy of LAC*::EV (left, strain RU129) and LAC*::*pamA* (right, strain RU121) biofilms after 24h of static growth. Magnification and scale bars indicated in yellow. **(B)** eDNA of LAC*::EV and LAC*::*pamA* in vitro biofilms and phosphate buffered saline (PBS) negative control after 24 h of static incubation, quantified by SYTOX Green eDNA staining. Data is pooled from three independent experiments with three biological replicates each (N=9 each strain). Statistical significance was determined by unpaired t-test, *****P*≤.0001. RFU, relative fluorescence unit.

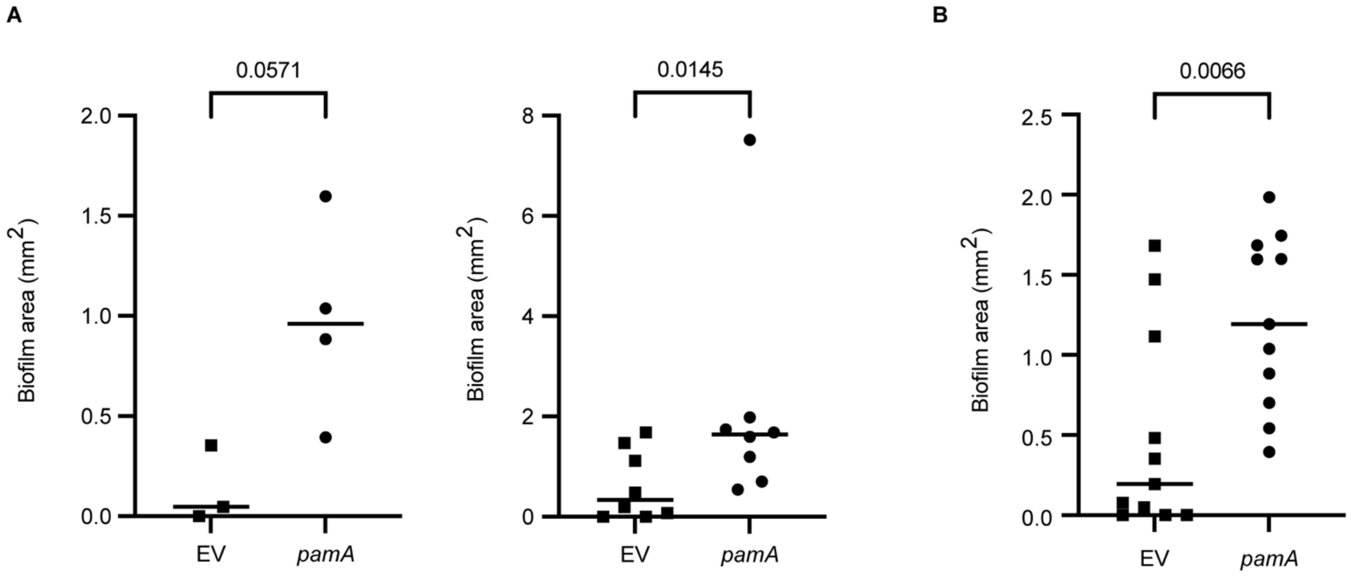

Supplemental Figure 9. Skin abscess biofilm data (Figure 6C) as independent replicate experiments and analysis with outlier removed. (A) The pooled data from **Figure 6C** presented as two independent experiments (experiment one, N=3-4 abscess per strain, left; experiment two, N=8 abscess per strain, right)**.** Biofilm area of LAC* containing *pamA* (strain RU121) or EV control (strain RU129) quantified as the difference between DAPI (total extracellular DNA) and 5mC (eukaryotic host extracellular DNA) staining (3). *P* value is annotated above the brackets, determined with the Mann-Whitney test. **(B)** Data from **Figure 6C** is presented with the outlier LAC*::*pamA* value (biofilm area 7.5 mm^2) omitted. The *P* value is annotated above the bracket, determined with the Mann-Whitney test. This panel shows that the results presented in **Figure 6C** retain statistical significance even with the outlier removed.

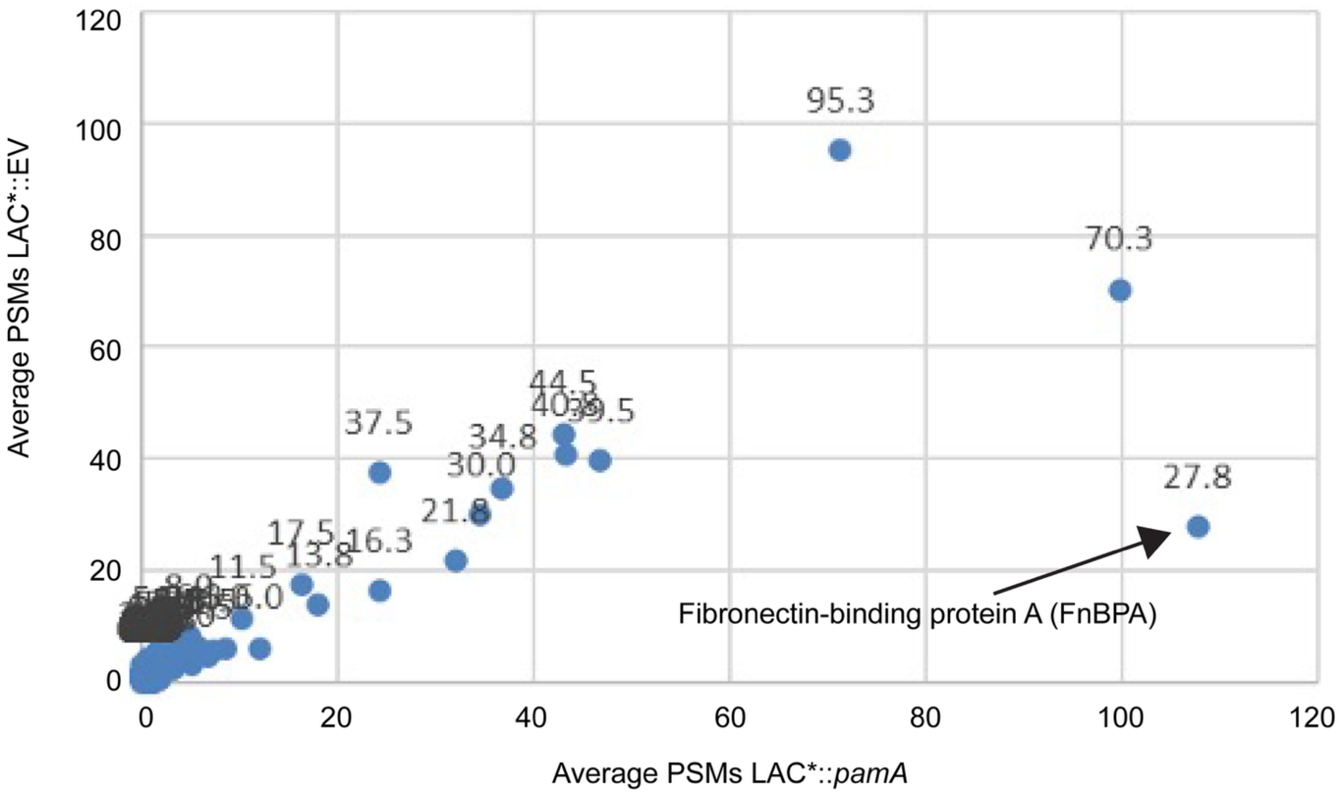

Supplemental Figure 10. Mass spectrometry suggests that FnBPA is the high molecular weight cell-wall-associated protein in LAC*::*pamA* biofilms. The high molecular weight band in the LAC*::*pamA* lanes **(Figure 6B)** and control areas of LAC*::EV lanes of similar molecular weight were extracted from SDS-PAGE gel and analyzed by mass spectrometry. The average peptide spectral matches (PSM) of LAC*::*pamA* (N=4 biological replicates, strain RU121) and LAC*::EV (N=4 biological replicates, strain RU129) are shown on the scatter plot. Data are pooled from two independent replicate experiments. The numbers above the data points correspond to the average PSMs in LAC*::EV. The data point representing FnBPA is highlighted with a black arrow.

**Start**	**End**	**Annotated product**	**Gene name**	**Motifs**
2056937	2057362	adenine-methyltransferase	ph11Mos_00044	Dam
2057606	2057767	Hypothetical protein	ph11Mos_00046	no domain
2057761	2058540	DNA replication protein DnaC	ph11Mos_00047	IstB_IS21
2058550	2059272	Helix-turn-helix protein	ph11Mos_00048	HTH_36
2059265	2059672	HNH endonuclease	ph11Mos_00049	HNH
2059727	2060491	AP2 domain protein	ph11Mos_00050	AP2
2062530	2062751	Hypothetical protein	ph11Mos_00054	DUF2483
2063564	2063794	Hypothetical protein	ph11Mos_00057	no domain

Supplemental Table 1. Candidate gene(s) for increased virulence localized by en bloc deletion experiments. Table adapted from (1) with start and stop positions, predicted annotations, gene name and predicted protein motifs of the mΦ11 ORFs that did not share homology with Φ11 within the candidate "virulence block" (Δ44-57) identified by en bloc deletion experiments (see Figure 1).

Supplemental Table 2: Bacterial Strains Used in this Study

Strain	Bacteria	Description	Brief Description	Citation
LAC*	*S. aureus*	USA300 LAC cured of *erm* resistance plasmid pUSA03 (clone AH1263), clonal complex (CC) 8	LAC*	(4)
BS989	*S. aureus*	LAC* lysogenized with mΦ11	LAC*/mΦ11	(1)
BS990	*S. aureus*	LAC* lysogenized with Φ11	LAC*/Φ11	(1)
RU42	*S. aureus*	LAC* lysogenized with mΦ11 containing deletion of genes 32-64	LAC*/mΦ11Δ32-64	This work
RU47	*S. aureus*	LAC* lysogenized with mΦ11 containing deletion of genes 32-43	LAC*/mΦ11Δ32-43	This work
RU108	*S. aureus*	LAC* lysogenized with mΦ11 containing deletion of 44-57	LAC*/mΦ11Δ44-57	This work
RU39	*S. aureus*	LAC* lysogenized with mΦ11 containing deletion of gene 44 (*pamA*)	LAC*/mΦ11Δ*pamA*	This work
IM08	*E. coli*	K-12, strain DC10B engineered to mimic the type I adenine methylation profile of *S. aureus* CC8. Available on BEI resources NR-49806.		(5)
RN4220	*S. aureus*	Restriction defective derivative of NCTC8325 cured of Φ11, Φ12, and Φ13		(6)

BS656	*S. aureus*	RN4220 with pRN7023 [shuttle vector containing SaPI1 *int*]		(7)
RU116	*S. aureus*	BS656 with *pamA* expression vector [pJC1111(P_{sarA}-*sod*RBS-*pamA*)] integrated at the SaPI1 site	BS656::pJC1111(P_{sarA}-*sod*RBS-*pamA*)	This work
RU120	*S. aureus*	BS656 with empty vector [pJC1111] integrated at the SaPI1 site	BS656::pJC1111	This work
RU121	*S. aureus*	LAC* with *pamA* expression vector [pJC1111(P_{sarA}-*sod*RBS-*pamA*)] integrated at the SaPI1 site	LAC*::pJC1111(P_{sarA}-*sod*RBS-*pamA*)	This work
RU128	*S. aureus*	LAC* with empty vector [pJC1111] integrated at the SaPI1 site and lysogenized with mΦ11 containing deletion of gene 44 (*pamA*)	LAC*/mΦ11Δ*pamA*::pJC1111	This work
RU129	*S. aureus*	LAC* with empty vector [pJC1111] integrated at the SaPI1 site	LAC*::pJC1111	This work
RU131	*S. aureus*	LAC* with *pamA* expression vector [pJC1111(P_{sarA}-*sod*RBS-*pamA*)] integrated at the SaPI1 site and lysogenized with mΦ11 containing deletion of gene 44 (*pamA*)	LAC*/mΦ11Δ*pamA*::pJC1111(P_{sarA}-*sod*RBS-*pamA*)	This work

RU138	*S. aureus*	LAC* with empty vector [pJC1111] integrated at the SaPI1 site and lysogenized with mΦ11	LAC*/mΦ11::pJC1111	This work
RU152	*S. aureus*	BS656 with *pamA* point-mutant P65A expression vector [pJC1111(P_{sarA}-*sod*RBS-*pamA*P65A)] integrated at the SaPI1 site	BS656::pJC1111(P_{sarA}-*sod*RBS-*pamA*P65A)	This work
RU154	*S. aureus*	BS656 with *pamA* point-mutant P65T expression vector [pJC1111(P_{sarA}-*sod*RBS-*pamA*P65A)] integrated at the SaPI1 site	BS656::pJC1111(P_{sarA}-*sod*RBS-*pamA*P65T)	This work
RU156	*S. aureus*	BS656 with *pamA* point-mutant P66A expression vector [pJC1111(P_{sarA}-*sod*RBS-*pamA*P65A)] integrated at the SaPI1 site	BS656::pJC1111(P_{sarA}-*sod*RBS-*pamA*P66A)	This work
RU161	*S. aureus*	LAC* with *pamA* point-mutant P65A expression vector [pJC1111(P_{sarA}-*sod*RBS-*pamA*P65A)] integrated at the SaPI1 site	LAC*::pJC1111(P_{sarA}-*sod*RBS-*pamA*P65A)	This work
RU162	*S. aureus*	LAC* with *pamA* point-mutant P65T expression vector [pJC1111(P_{sarA}-*sod*RBS-*pamA*P65A)] integrated at the SaPI1 site	LAC*::pJC1111(P_{sarA}-*sod*RBS-*pamA*P65T)	This work
RU164	*S. aureus*	LAC* with *pamA* point-mutant P66A expression vector [pJC1111(P_{sarA}-*sod*RBS-*pamA*P65A)] integrated at the SaPI1 site	LAC*::pJC1111(P_{sarA}-*sod*RBS-*pamA*P66A)	This work
NE186	*S. aureus*	USA300 strain JE2 with transposon insertion (disrupted by *bursa aurealis*) in *fnbA*	JE2, *fnbA::bursa*	(8)

RU169	*S. aureus*	LAC* with *pamA* expression vector [pJC1111(P_{sarA}-*sod*RBS-*pamA*)] integrated at the SaPI1 site and transposon mutagenesis of *fnbA*	LAC*::pJC1111(P_{sarA}-*sod*RBS-*pamA*)+*fnbA::bursa*	This work
RU170	*S. aureus*	LAC* with empty vector [pJC1111] integrated at the SaPI1 site and transposon mutagenesis of *fnbA*	LAC*::pJC1111+*fnbA::bursa*	This work
RU171	*S. aureus*	LAC* lysogenized with mΦ11 and transposon mutagenesis of *fnbA*	LAC*/mΦ11+*fnbA::bursa*	This work
BVED028	*S. aureus*	MRSA skin infection isolate strain, CC1 background (*spa* type t1508), Panton-Valentine leukocidin (PVL) positive		This work
RU241	*S. aureus*	BVED028 with *pamA* expression vector [pJC1111(P_{sarA}-*sod*RBS-*pamA*)] integrated at the SaPI1 site	BVED028::pJC1111 (P_{sarA}-*sod*RBS-*pamA*)	This work
RU242	*S. aureus*	BVED028 with empty vector [pJC1111] integrated at the SaPI1 site	BVED028::pJC1111	This work

Supplemental Table 3. Plasmids, Oligonucleotides, and Probes Used in this Study

**Plasmid**	**Resistance**	**Description**	**Citation**
pIMAY	Cm	Allelic exchange plasmid for *S. aureus*; pIMC5 with tetracycline and inducible *secY* antisense from pKOR1	(9)
pRU1	Cm	pIMAY with *pamA* deletion insert (591 bp upstream *pamA* and 600 bp downstream of *pamA*)	This work
pRU2	Cm	pIMAY with Δ32-64 deletion insert (985 bp upstream mΦ11 gene 32 and 969 downstream mΦ11 gene 64)	This work
pRU3	Cm	pIMAY with Δ32-43 deletion insert (985 bp upstream mΦ11 gene 32 and 995 downstream mΦ11 gene 43)	This work
pRU4	Cm	pIMAY with Δ44-57deletion insert (985 bp upstream *pamA* and 995 bp downstream of mΦ11 gene 57)	This work
pOS1-P_{sarA}-*sod*RBS-GFP	Amp (*E. Coli*), Erm (*S. aureus*)	pOS1 containing *sarA* promoter, *sod* ribosome binding site, and GFP (also called pOS1sGFP)	(10)
pJC1111	Cd	SapI1 *attS* suicide vector	(11)
pRU7	Cd	pJC1111(P_{sarA}-*sod*RBS-*pamA*)	This work
pRU9	Cd	pJC1111(P_{sarA}-*sod*RBS-*pamA*P65A)	This work
pRU10	Cd	pJC1111(P_{sarA}-*sod*RBS-*pamA*P65T)	This work
pRU11	Cd	pJC1111(P_{sarA}-*sod*RBS-*pamA*P66A)	This work
**Oligonucleotide**	**Direction**	**5'-3' Sequence**	**Citation**

oRU1	fwd	cctcgaggtcgacggtatcgataagcttGTCATACTCTAGTATTTCGT CTGGATTG	This work
oRU2	rev	TTTCCTAGCGCAATTATCGTTTATAGA	This work
oRU3	fwd	tctataaacgataattgcgctaggaaaACTGTAATGTACTTCCATGTG CC	This work
oRU4	rev	tggcggccgctctagaactagtggatccCAGCATTGTTGATAACAGAG TAGGT	This work
oRU5	fwd	cctcgaggtcgacggtatcgataagcttAAACGCAGTACGATAGTCAA TATCC	This work
oRU6	rev	GATACAGGAGATGACAATGATGATTAAAC	This work
oRU7	fwd	gtttaatcatcattgtcatctcctgtatcATGGTATCATATCGGTATCAAA TAACGATT	This work
oRU8	rev	tggcggccgctctagaactagtggatccCGAAATCAGTTACGATTACA TTACCTGT	This work
oRU9	fwd	gtttaatcatcattgtcatctcctgtatcACTCATTGTGCACCTCTATAAA CG	This work
oRU10	fwd	tctataaacgataattgcgctaggaaaCAGTTAAATATCTTTTAGATC GTGACTAGC	This work
oRU11	rev	tggcggccgctctagaactagtggatccGGAAGCCCAAATGCAAGAA TTAC	This work

oRU15	fwd	AAGCTTATCGATACCGTCGACCTCGAGG	This work
oRU16	rev	GGATCCACTAGTTCTAGAGCGGCCGCCA	This work
oRU17	fwd	cctcgaggtcgacggtatcgataagcttCCTGCCATTTCTTAAACATTTGGTTATAA	This work
oRU41	fwd	GCTGGCGGCCGCTGCATGCCTGCAG	This work
oRU42	rev	ATTCGAGCTCGGTACCCGGGGATCC	This work
oRU43	fwd	gctggcggccgctgcatgcctgcagCTGATATTTTTGACTAAACCAAATGC	This work
oRU44	rev	CTACTGTAATGTACTTCCATAAATAATCATCCTCCTAAGGTACC	This work
oRU45	fwd	CCTTAGGAGGATGATTATTTATGGAAGTACATTACAGTAGTAAAAC	This work
oRU46	rev	attcgctcggtacccggggatccTCATTGTGCACCTCTATAAACGag	This work
oRU70	fwd	GACCGTATGGCGCGTTCATAAAAAC	This work
oRU71	rev	GTTTTTATGAACGCGCCATACGGTC	This work
oRU72	fwd	GACCGTATGGCGTGTTCATAAAAAC	This work
oRU73	rev	GTTTTTATGAACACGCCATACGGTC	This work
oRU74	fwd	GACCGTATGCCGGGTTCATAAAAAC	This work
oRU75	rev	GTTTTTATGAACCCGGCATACGGTC	This work
oRU80	fwd	CATGTTCTTTCCTGCGTTATCC	This work
oRU81	rev	GCATTTAGAATAGGCGCGC	This work
oRU84	fwd	GAGATTGTGTTGTTTCCTTAAC	This work
oRU85	rev	GGGTTTCTGATGACTTGAATA	This work

IM151	fwd	TACATGTCAAGAATAAACTGCCAAAGC	(9)
IM152	rev	AATACCTGTGACGGAAGATCACTTCG	(9)
**qRT-PCR target**	**Direction**	**5’-3’ Sequence**	**Citation**
rpoB	fwd	GAACATGCAACGTCAAGCAG	(12)
rpoB	rev	AATAGCCGCACCAGAATCAC	(12)
rpoB	probe	HEX/TACAGGTATGGAACACGTTGCAGCA/BHQ_1	(12)
pamA	fwd	GACTGGTCTGAGGACATTGTTT	This work
pamA	rev	GTGTCTGTTCTTGCGGGTATTA	This work
pamA	probe	HEX/TTATGAACCCGCCATACGGTCGAA/BHQ_1	This work
fnbA	fwd	GTCCTGCATGAGGTTCTACTTT	This work
fnbA	rev	CAGATGTAGCGGAAGCTAAGG	This work
fnbA	probe	5HEX/ACCCGTTTC/ZEN/CACTTTCGCGTTACT/3IABkFQ	This work

Supplemental Methods

A. Construction of bacterial strains

Construction of en bloc and pamA deletion mutants. In-frame, unmarked, deletions were engineered by amplifying 600-1000 base pair fragments flanking the target gene(s) using genomic DNA (gDNA) template from strain LAC*/mΦ11 (BS989) and the following oligonucleotide pairs [oRU1/oRU2 (upstream) and oRU3/oRU4 (downstream) to construct pRU1 for LAC*/mΦ11Δ*pamA* (RU39); oRU5/oRU6 (upstream) and oRU7/oRU8 (downstream) to construct pRU2 for LAC*/mΦ11Δ32-64 (RU42); oRU5/oRU6 (upstream) and oRU9/oRU4 (downstream) to construct pRU3 for LAC*/mΦ11Δ32-43 (RU47); oRU17/oRU2 (upstream) and oRU10/oRU11 (downstream) to construct pRU4 for LAC*/mΦ11Δ44-57 (RU108)] **(Supplemental Table 3)**. Amplified fragments were purified with PCR clean-up kit (Qiagen, #28104) and inserted into an inverse PCR amplified (oRU15/oRU16) pIMAY cloning plasmid (9) using Gibson assembly (New England Biolabs, #E2611S). Next, 5 µL of Gibson product was added to 50µL of chemically competent *E. coli* IM08, incubated 30 min on ice, heat shocked in 42°C water for 45s, then 950 µL LB was added for recovery incubation at 37°C with shaking at 250 rpm. After recovery, 100 µL of sample was plated on prewarmed LB plates with Cm and incubated at 37°C. Plasmids pRU1, pRU2, pRU3 and pRU4 were isolated from colonies using QIAprep Spin Miniprep kit (Qiagen, #27106). The cloning inserts were validated by Sanger sequencing (Psomagen, Inc.) of PCR amplification product using primers IM151/IM152 and the plasmids were electroporated into electrocompetent strain LAC*/mΦ11 (BS989) for allelic exchange using established protocols (9).

Chromosomal insertion of constitutively expressed pamA. For single-copy chromosomal insertion, a fragment containing the constitutive *sarA* promoter and *sod* ribosome binding site

(P_{sarA}-*sod*RBS) was PCR amplified using DNA template from pOS1-P_{SarA}-*sod*RBS-GFP (10) and oligonucleotide pair oRU43/oRU44, and a fragment containing the full-length *pamA* gene was PCR amplified using gDNA template from strain BS989 and oligonucleotide pair oRU45/oRU46. The fragments were separated on 1% agarose gel and isolated and purified with QIAquick gel extraction kit (Qiagen, #28704) per manufacturers protocol. pJC1111, a suicide plasmid with Amp and Cd resistance markers that integrates at the *S. aureus* pathogenicity island 1 (SapI1) *att* site (7), was PCR amplified using oligonucleotide pair oRU41/oRU42 and the P_{sarA}-*sod*RBS and *pamA* amplification products were ligated into pJC1111 using Gibson assembly, generating pRU7. Transformants were selected on LB/Amp plates and validated by sequencing using primers oRU80/oRU81. Once the insert was confirmed, pRU7 was isolated using a QIAprep Spin Miniprep kit (Qiagen, #27106) and transformed into strain BS656 for chromosomal integration using published protocols (7), generating strain RU116. To generate control strains, pJC1111 was transformed into strain BS656 for chromosomal integration, generating strain RU120. Chromosomal integrants at the SapI1 chromosomal position were selected with $CdCl_2$. Phage 80α lysates of RU116 were used to transduce LAC*, RU39, and BVED028, generating RU121, RU131, and RU241 respectively. Phage 80α lysates of RU120 were used to transduce LAC*, RU39, BS989, and BVED028 generating empty vector (EV) control strains RU129, RU128, RU138, and RU242 respectively. The P_{sarA}-*sod*RBS-*pamA* and EV inserts were validated by PCR amplification and Sanger sequencing using primers oRU80/oRU81.

Chromosomal insertion of constitutively expressed pamA point mutants. Point mutants were created using complementary oligonucleotides engineered to contain the desired *pamA* point mutation [oRU70/oRU43 (upstream) and oRU71/oRU46 (downstream) to construct pRU9 for *pamA*P65A strain RU161; oRU72/oRU43 (upstream) and oRU73/oRU46 (downstream) to

construct pRU10 for *pamA*P65T mutant strain RU162; oRU74/oRU43 (upstream) and oRU75/oRU46 (downstream) to construct pRU11 for PamAP66A mutant (strain RU164)] to amplify fragments from pJC1111::P_{sarA}-*sod*RBS-*pamA* template DNA. The fragments were gel purified using QIAquick gel extraction kit (Qiagen, #28704) and joined with overlap extension (OE) PCR (13) using oligonucleotide pair oRU43/oRU46. After OE-PCR, the fragments were inserted into pJC1111 using Gibson assembly, transformed into competent *E. Coli* DH5α (New England Biolabs, #C2987H) per manufacturer instructions and plated on LB/Amp plates. Colony PCR (primers oRU80/oRU81) of colonies growing on LB/Amp plates confirmed the insert and the product was sent for Sanger sequencing. Once the desired point-mutated insert was confirmed, pRU9, pRU10, and pRU11 were harvested from *E. Coli* DH5α using Qiagen miniprep kit, electroporated into competent *S. aureus* strain BS656 (resulting in strains RU152, RU154, and RU156, respectively), and transduced into strain LAC* using phage 80α (resulting in strains RU161, RU162, and RU164, respectively). Transductants were selected on TSA/Cd plates, underwent colony PCR using oligonucleotide pair oRU80/oRU81 and Sanger sequencing of the amplified product confirmed the desired point mutation. The inactivation of *pamA* was confirmed with DpnI digestion as described below.

Fibronectin-binding protein A (fnbA) transposon mutants. Phage 80α lysate of Nebraska Transposon Mutant Library (8) strain NE186 (*fnbA*::*bursa,* Erm) was used to transduce RU121, RU129, and BS989, to generate RU169, RU170, and RU171, respectively. PCR amplification using primers to amplify the *fnbA* transposon insertion site (oRU84/oRU85) confirmed the insertion.

B. Protein identification by mass spectrometry

After digestion, the solution containing the peptides was transferred into a new Eppendorf tube and the gels washed via shaking with a 1:2 (vol/vol) 5% formic acid/ acetonitrile extraction buffer for 15 min at 37°C. The extraction buffer was removed and combined with the previous aspirated solution and the process repeated two more times. The combined solutions were dried using a SpeedVac (Thermo Savant). The dried sample was reconstituted in 0.1% TFA and desalted using C18 microspin columns (Harvard apparatus (Millipore)). The bound peptides were rinsed three times with 0.1% TFA and one time with 0.5% acetic acid and elute off the microspin column using 40 μL 40% acetonitrile in 0.5% acetic acid followed by the addition of 40 μL 80% acetonitrile in 0.5% acetic acid. The organic solvent was removed using a SpeedVac concentrator and the sample was reconstituted in 0.5% acetic acid. For mass spectrometry (MS) analysis, an aliquot of each sample (1/10) was loaded onto an Acclaim PepMap trap column (2 cm x 75 μm) in line with an EASY-Spray analytical column (50 cm x 75 μm ID PepMap C18, 2 μm bead size) using the autosampler of an EASY-nLC 1200 HPLC (Thermo Scientific). Solvent A was 2% acetonitrile in 0.5% acetic acid and solvent B was 80% acetonitrile in 0.5% acetic acid. The sample was gradient eluted in the Orbitrap Eclipse (Thermo Fisher). The gradient was held for 5 min at 5% solvent B, ramped in 60 min to 35% solvent B, in 10 min to 45% solvent B, and in another 10 min to 100% solvent B. High resolution full MS spectra were acquired with a resolution of 120,000, an AGC target of 4e5, with a maximum ion time of 50 ms, and scan range of 400 to 1500 m/z. Following each full MS scan, precursor ions with charge states between 2-5 were considered for fragmentation for a cycle duration of 3 seconds. MS/MS HCD spectra were collected with a resolution of 30,000, an AGC target of 2e5, maximum injection time of 200 ms, one microscan, 2 m/z isolation window, fixed first mass of 150, and normalized collision energy (NCE) of 27. Dynamic exclusion was set to 30 seconds. The MS/MS spectra were searched against a *S. aureus* USA300 database and common

contaminants (cRAP) using Sequest within Proteome Discoverer 1.4. using the following settings: oxidized methionine (M), and deamidation (asparagine and glutamine) were selected as variable modifications, and carbamidomethyl (C) as fixed modifications; precursor and fragment mass tolerance was set to 10 ppm. The data was filtered to better than 1% peptide and protein FDR searched against a decoy database. Only proteins with at least two different peptides were considered for downstream analysis.

Supplemental References

1. Copin R, Sause WE, Fulmer Y, Balasubramanian D, Dyzenhaus S, Ahmed JM, et al. Sequential evolution of virulence and resistance during clonal spread of community-acquired methicillin-resistant Staphylococcus aureus. *Proc Natl Acad Sci U S A.* 2019.
2. Quinlan AR, and Hall IM. BEDTools: a flexible suite of utilities for comparing genomic features. *Bioinformatics.* 2010;26(6):841-2.
3. Lacey KA, Serpas L, Makita S, Wang Y, Rashidfarrokhi A, Soni C, et al. Secreted mammalian DNases protect against systemic bacterial infection by digesting biofilms. *J Exp Med.* 2023;220(6).
4. Boles BR, Thoendel M, Roth AJ, and Horswill AR. Identification of genes involved in polysaccharide-independent Staphylococcus aureus biofilm formation. *PLoS One.* 2010;5(4):e10146.
5. Monk IR, Tree JJ, Howden BP, Stinear TP, and Foster TJ. Complete Bypass of Restriction Systems for Major Staphylococcus aureus Lineages. *mBio.* 2015;6(3):e00308-15.
6. Novick RP. Genetic systems in staphylococci. *Methods Enzymol.* 1991;204:587-636.

7. Chen J, Yoong P, Ram G, Torres VJ, and Novick RP. Single-copy vectors for integration at the SaPI1 attachment site for Staphylococcus aureus. *Plasmid.* 2014;76:1-7.

8. Fey PD, Endres JL, Yajjala VK, Widhelm TJ, Boissy RJ, Bose JL, et al. A genetic resource for rapid and comprehensive phenotype screening of nonessential Staphylococcus aureus genes. *mBio.* 2013;4(1):e00537-12.

9. Monk IR, Shah IM, Xu M, Tan MW, and Foster TJ. Transforming the untransformable: application of direct transformation to manipulate genetically Staphylococcus aureus and Staphylococcus epidermidis. *MBio.* 2012;3(2).

10. Benson MA, Lilo S, Wasserman GA, Thoendel M, Smith A, Horswill AR, et al. Staphylococcus aureus regulates the expression and production of the staphylococcal superantigen-like secreted proteins in a Rot-dependent manner. *Mol Microbiol.* 2011;81(3):659-75.

11. Geisinger E, George EA, Chen J, Muir TW, and Novick RP. Identification of ligand specificity determinants in AgrC, the Staphylococcus aureus quorum-sensing receptor. *J Biol Chem.* 2008;283(14):8930-8.

12. Anderson EE, Dyzenhaus S, Ilmain JK, Sullivan MJ, van Bakel H, and Torres VJ. SarS Is a Repressor of Staphylococcus aureus Bicomponent Pore-Forming Leukocidins. *Infect Immun.* 2023;91(4):e0053222.

13. Thornton JA. Splicing by Overlap Extension PCR to Obtain Hybrid DNA Products. *Methods Mol Biol.* 2016;1373:43-9.
