# Supplementary material for: Prophage-encoded methyltransferase drives adaptation of community-acquired methicillin-resistant Staphylococcus aureus
Source: J Clin Invest. 2025 Jul 22;135(18):e177872. doi: 10.1172/JCI177872 (PMC12435837; doi:10.1172/JCI177872)

Uncropped Gels

Figure 4B. Effect of PamA point mutants on methylase activity. The area included in the figure is highlighted by yellow box.

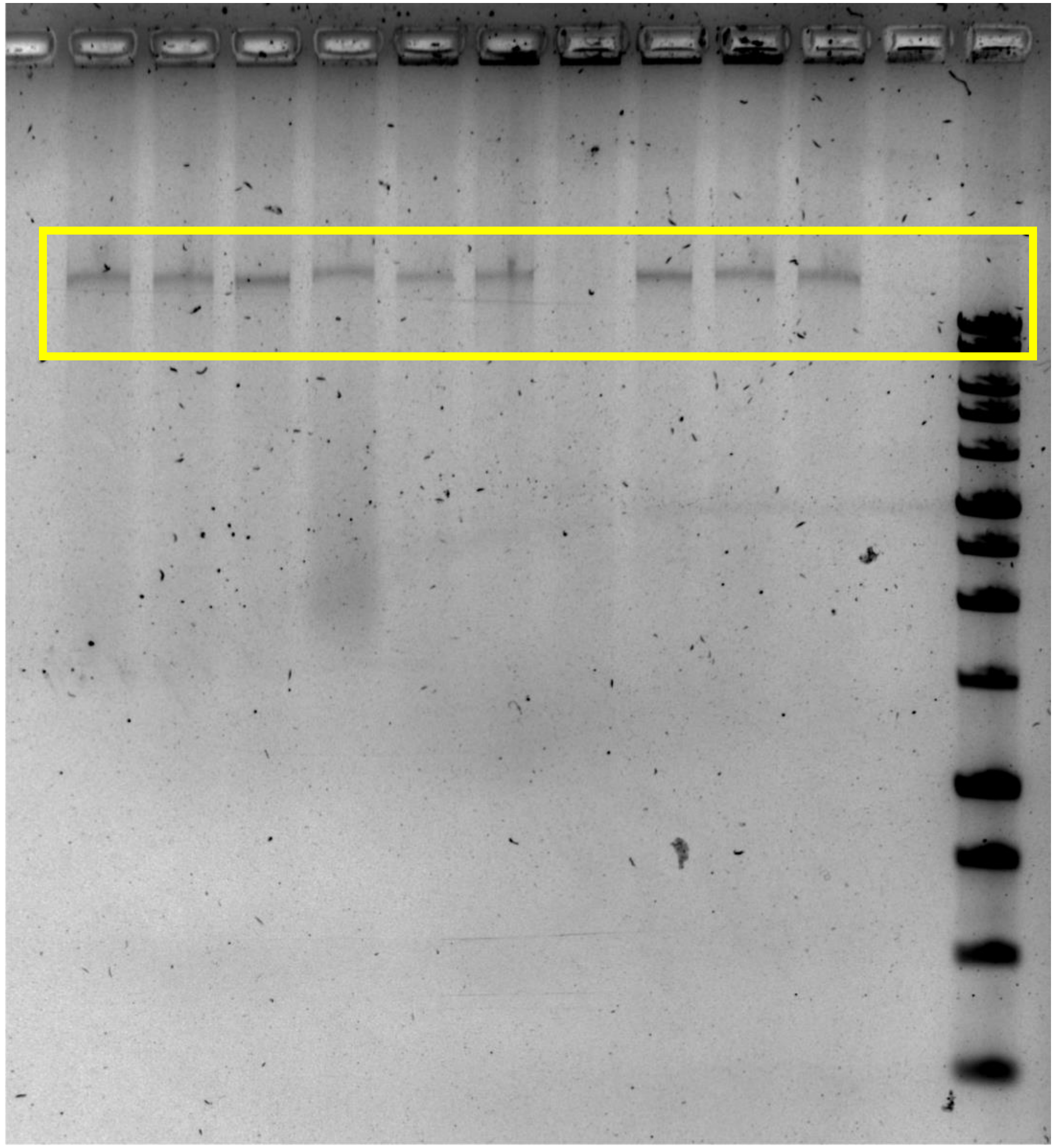

Figure 6D. cell wall associated proteins from biofilm of EV (N=3 bio replicates, left 3 wells) and pamA (N=3 bio replicates, right 3 lanes). The included area is highlighted with a yellow box.

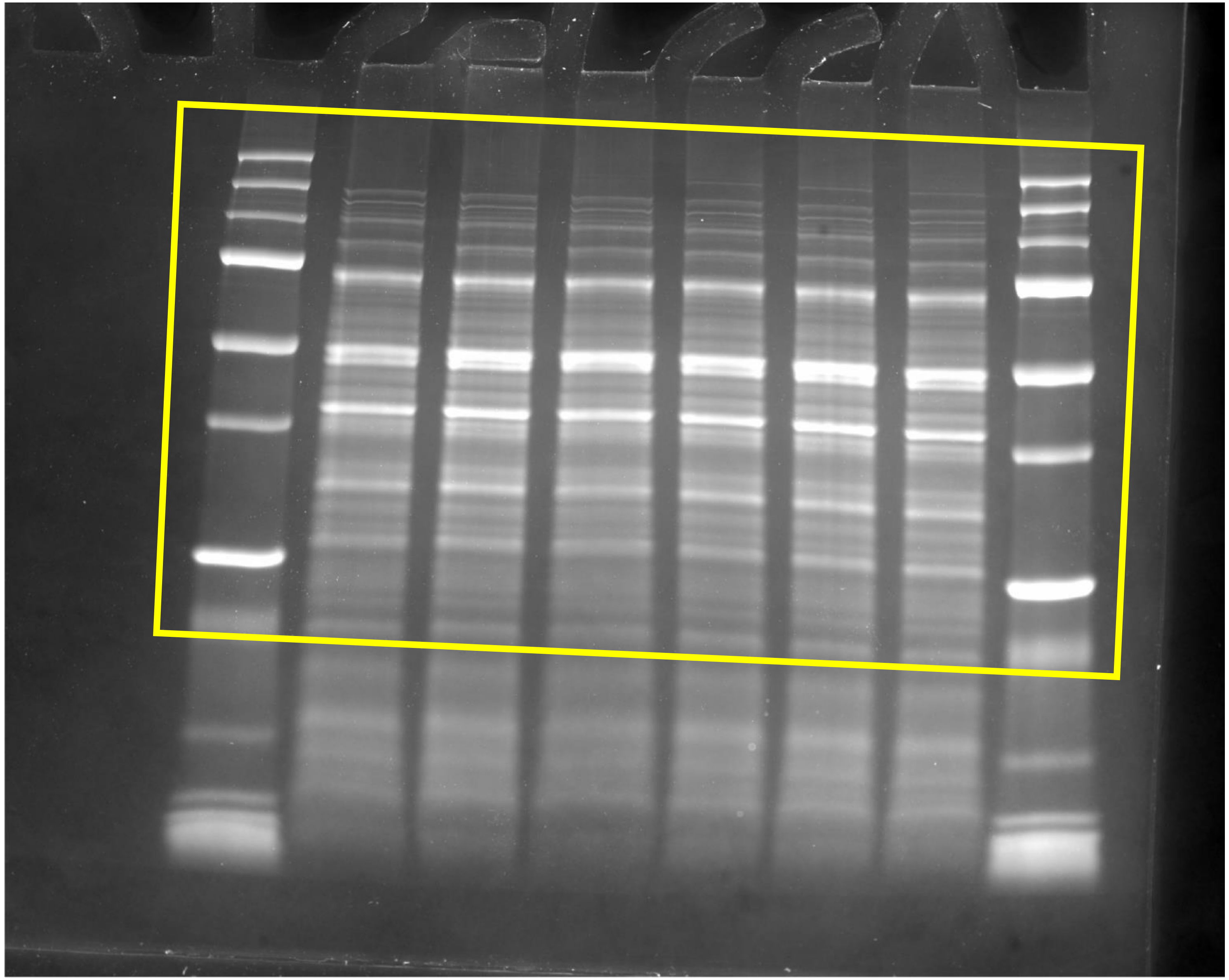

Figure 6E. Western Blot of cell wall associated proteins from biofilm of EV (N=3 bio replicates) and pamA (N=3 bio replicates). Coomassie gel of this western is shown Fig 6D. Of note, the ~70kb band across all strains is non-specific binding to staphylococcal protein A (Spa), see uncropped gel for Fig 6G. The area included in the figure is highlighted by yellow box.

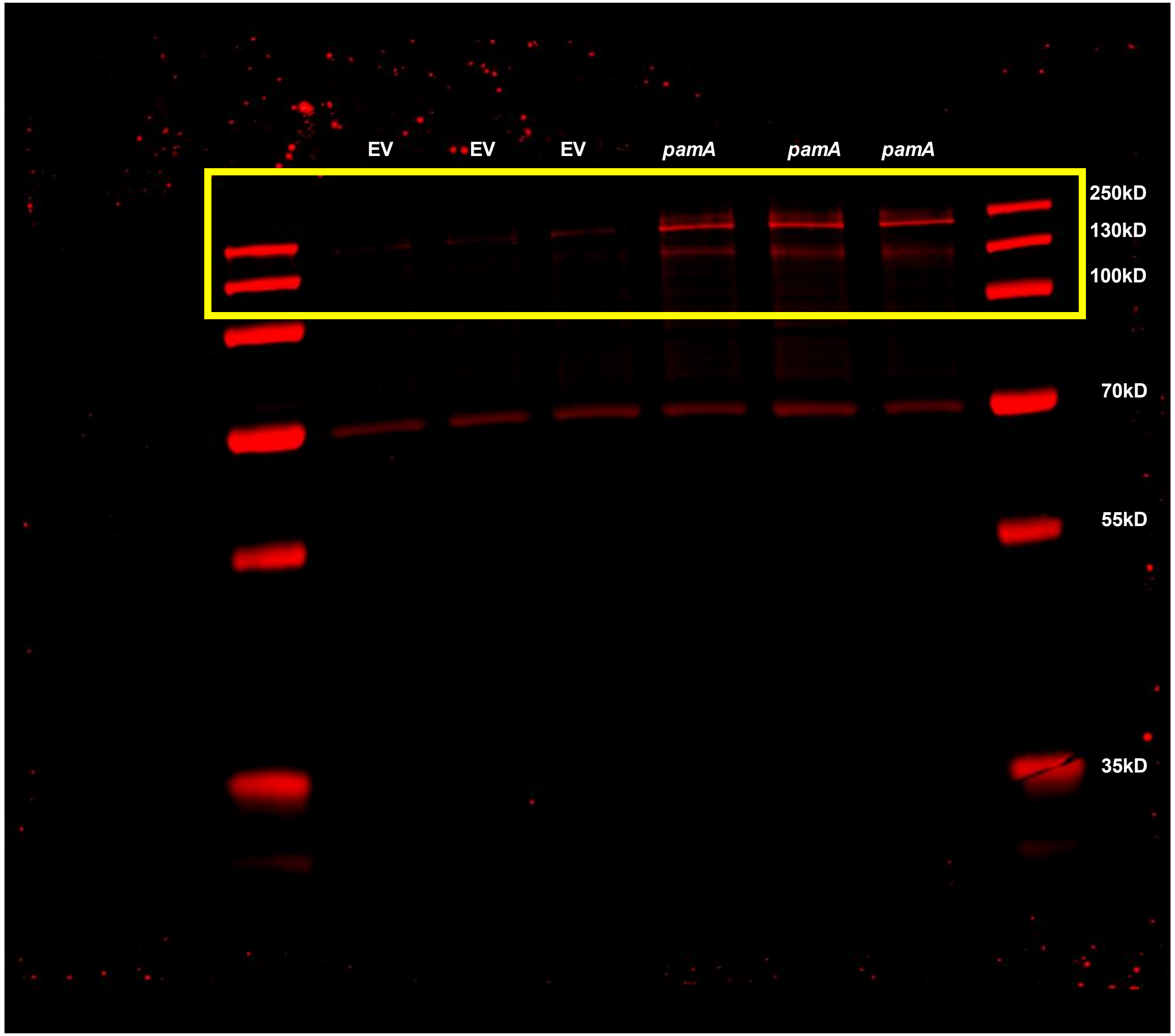

Figure 6G. Western Blot of cell wall associated proteins from biofilms of EV, pamA, pamA+fnbA::bursa and EV△spa△sbi. Two biological replicates shown on left and right side of gel. The area included in the figure is highlighted by yellow box.

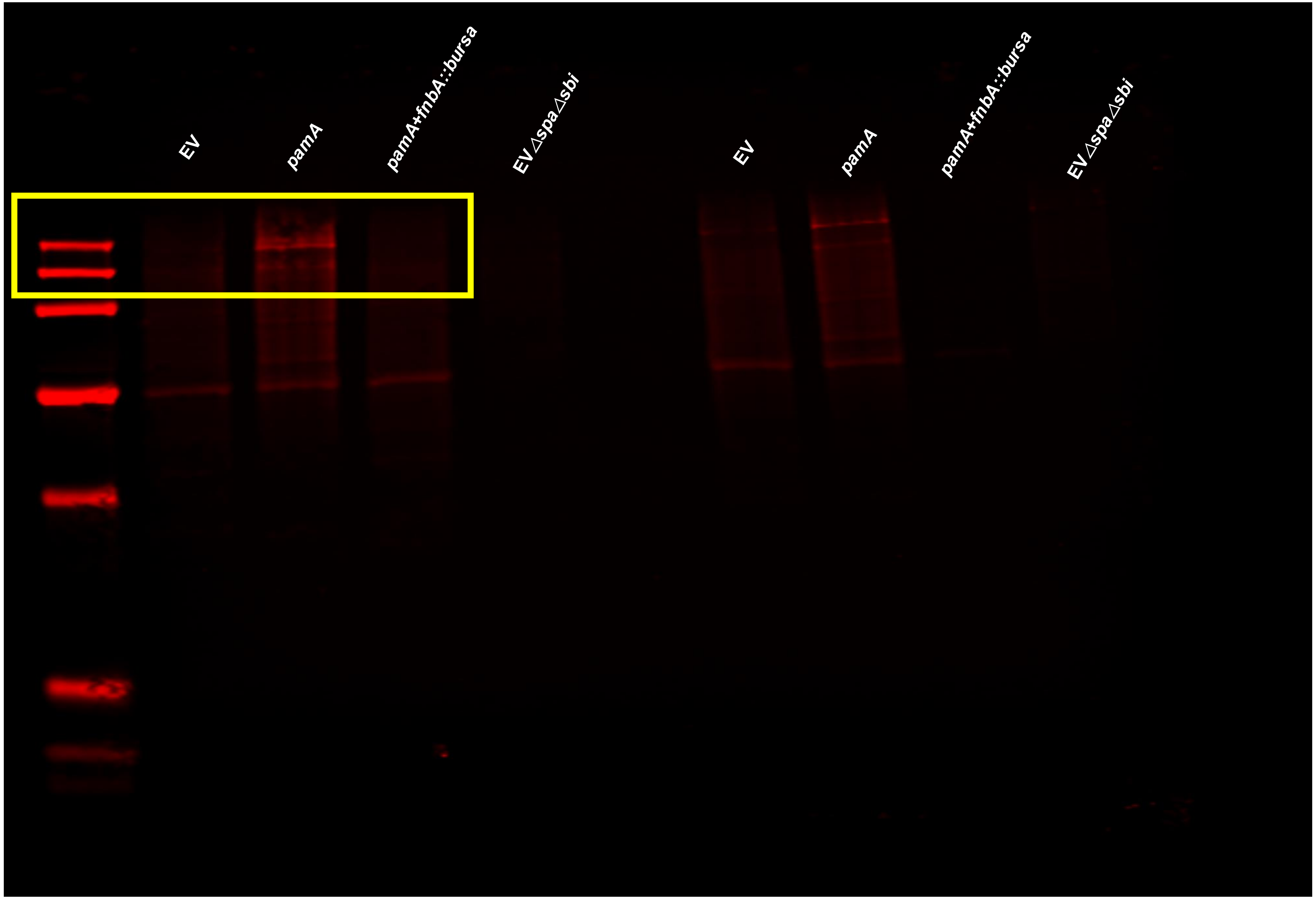

Supplemental Figure 1E. Exoproteins after 6h (left) and 24h (right) planktonic growth. The area included in the figure is highlighted by yellow boxes.

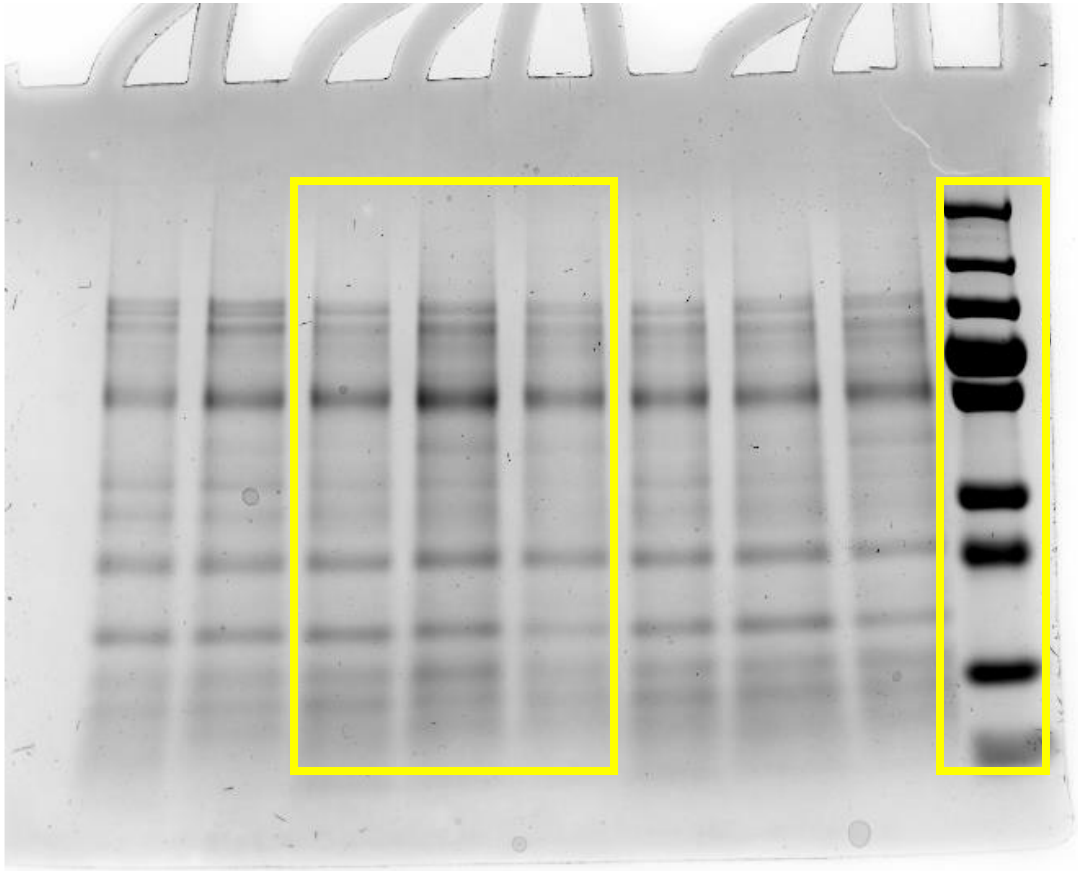

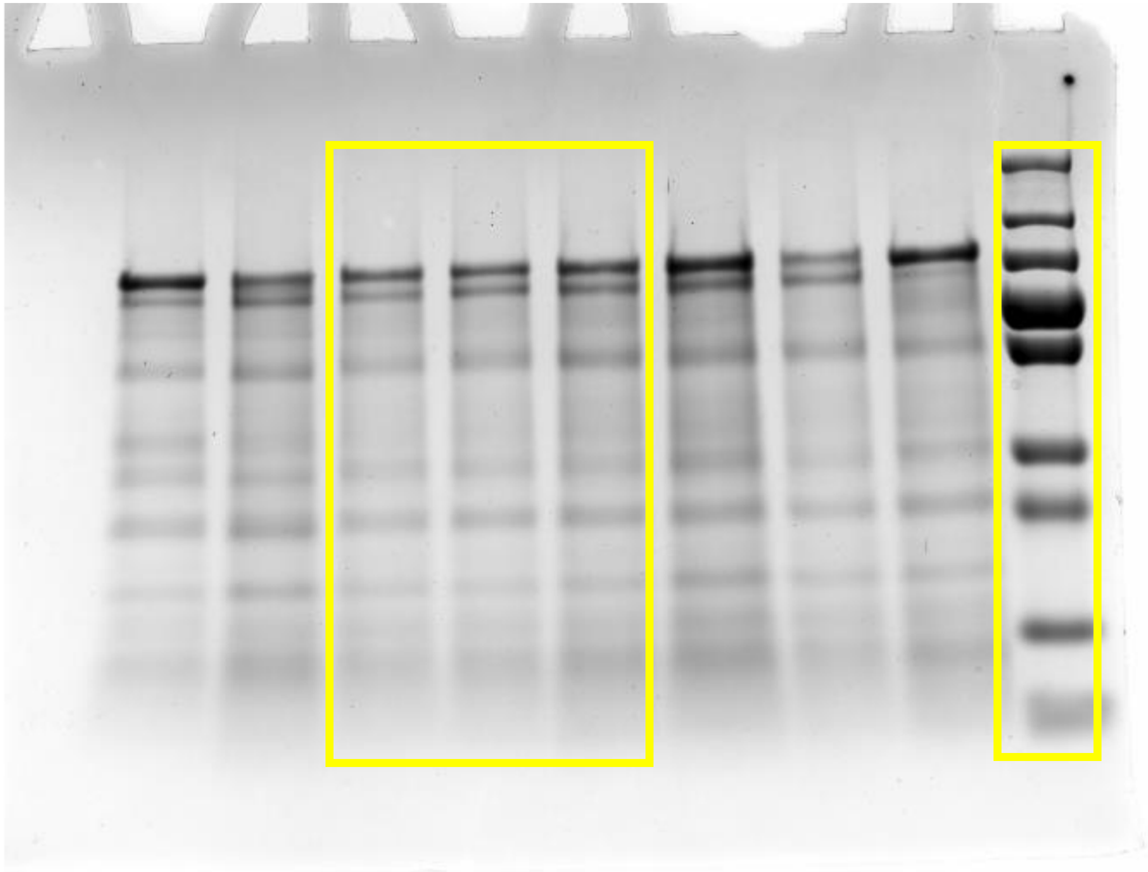

Supplemental Figure 7. DpnI digestion of parental and *pamA* expression vector strains after in vitro growth. The area included in the figure is highlighted by yellow box. The omitted area (right side of gel) was the negative control using the same strains, showing no digestion of gDNA in any strains without DpnI.

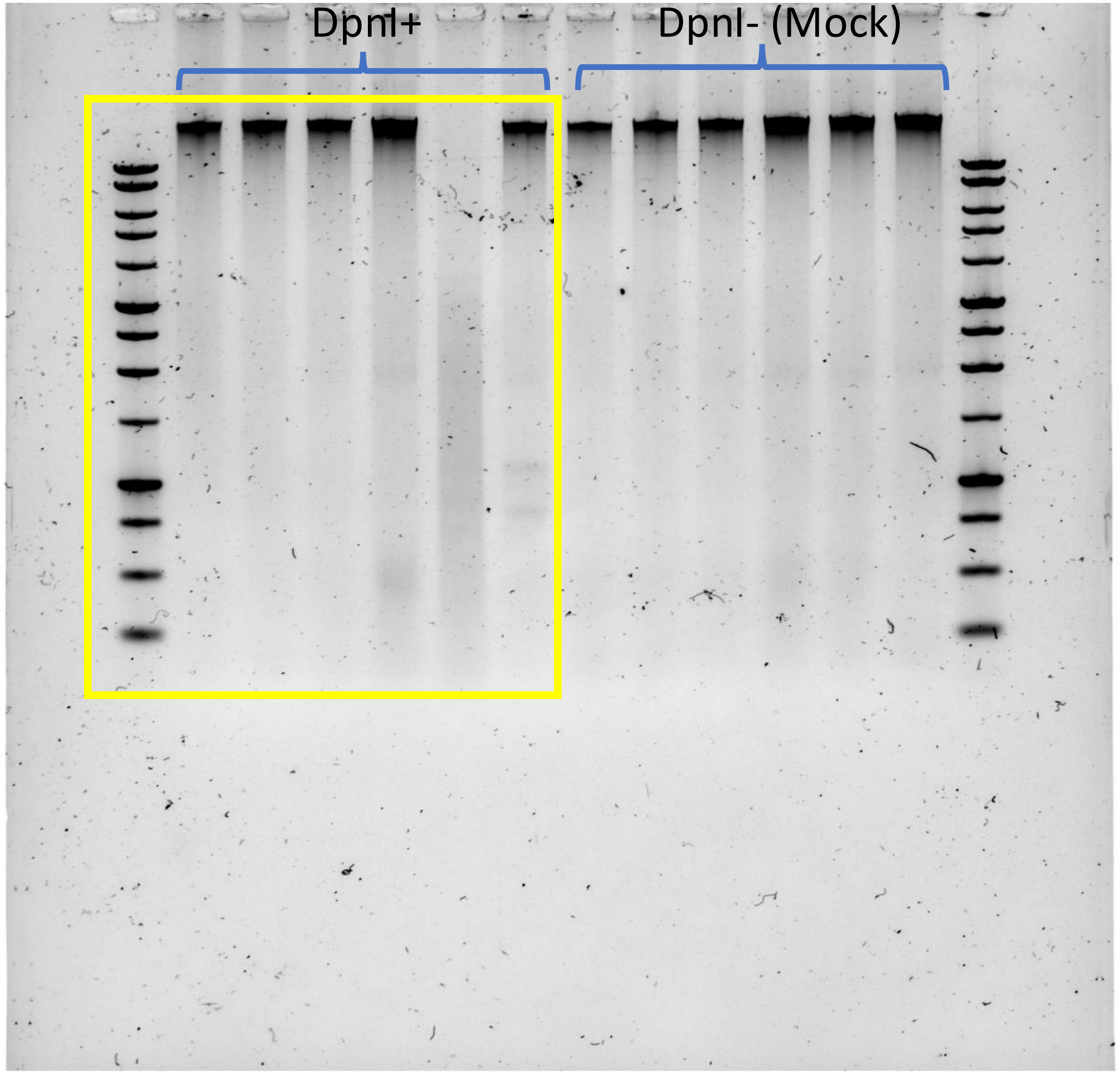

Supplement: Unedited blot and gel images [file jci-135-177872-s058.pdf]
